# Stochastic scanning events on the GCN4 mRNA 5’ untranslated region generate cell-to-cell heterogeneity in the yeast nutritional stress response

**DOI:** 10.1093/nar/gkad433

**Published:** 2023-05-29

**Authors:** Xiang Meng, Alan Reed, Sandie Lai, Juraj Szavits-Nossan, John E G McCarthy

**Affiliations:** Warwick Integrative Synthetic Biology Centre (WISB) and School of Life Sciences, University of Warwick, Gibbet Hill, Coventry CV4 7AL, UK; Warwick Integrative Synthetic Biology Centre (WISB) and School of Life Sciences, University of Warwick, Gibbet Hill, Coventry CV4 7AL, UK; Warwick Integrative Synthetic Biology Centre (WISB) and School of Life Sciences, University of Warwick, Gibbet Hill, Coventry CV4 7AL, UK; School of Biological Sciences, University of Edinburgh, Edinburgh EH9 3JH, UK; Warwick Integrative Synthetic Biology Centre (WISB) and School of Life Sciences, University of Warwick, Gibbet Hill, Coventry CV4 7AL, UK

## Abstract

Gene expression stochasticity is inherent in the functional properties and evolution of biological systems, creating non-genetic cellular individuality and influencing multiple processes, including differentiation and stress responses. In a distinct form of non-transcriptional noise, we find that interactions of the yeast translation machinery with the *GCN4* mRNA 5’UTR, which underpins starvation-induced regulation of this transcriptional activator gene, manifest stochastic variation across cellular populations. We use flow cytometry, fluorescence-activated cell sorting and microfluidics coupled to fluorescence microscopy to characterize the cell-to-cell heterogeneity of *GCN4*-5’UTR-mediated translation initiation. *GCN4-*5’UTR-mediated translation is generally not de-repressed under non-starvation conditions; however, a sub-population of cells consistently manifests a stochastically enhanced *GCN4* translation (SET*^GCN4^*) state that depends on the integrity of the *GCN4* uORFs. This sub-population is eliminated upon deletion of the Gcn2 kinase that phosphorylates eIF2α under nutrient-limitation conditions, or upon mutation to Ala of the Gcn2 kinase target site, eIF2α-Ser51. SET*^GCN4^* cells isolated using cell sorting spontaneously regenerate the full bimodal population distribution upon further growth. Analysis of *ADE8::ymRuby3*/ *GCN4::yEGFP* cells reveals enhanced Gcn4-activated biosynthetic pathway activity in SET*^GCN4^* cells under non-starvation conditions. Computational modeling interprets our experimental observations in terms of a novel translational noise mechanism underpinned by natural variations in Gcn2 kinase activity.

## Graphical Abstract

**Graphical Abstract:**
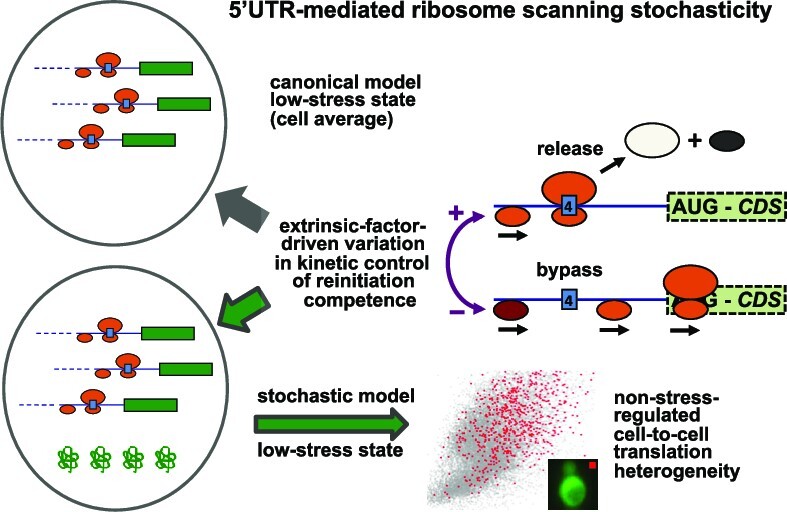

## INTRODUCTION

Gene expression stochasticity underpins a wide range of phenomena that are critical to organism functionality and viability, including cellular auto-regulatory circuits, phenotypic variation, differentiation, stress responses, synchrony in circadian clocks, and probabilistic fate decisions such as viral latency (1–8). These various lines of evidence point to a major role for noise in evolution (9–12). At the same time, other reports have revealed that noise is a potentially damaging source of imprecision, for example impacting on signaling and regulation (13–16). In response to this threat of disorder, living systems use multiple mechanisms to keep randomness under control. Overall, understanding gene expression noise, and the mechanisms used in living systems to manage it, is essential to achieving a complete understanding of biology. Such knowledge also provides important guiding principles for the design and engineering of biological systems.

Gene expression noise is generally categorized in two different components: intrinsic noise that is attributed to inherent stochasticity of expression from a specified gene system, and extrinsic noise that results from fluctuations in the intracellular environment, for example linked to the cell cycle and/or changes in the capacity of the expression machinery (17–19). It has previously been observed that total noise squared (*η_tot_*^2^) equates to the sum of the intrinsic and extrinsic noise components $\eta _{{\mathop{\rm int}} }^2 + \eta _{ext}^2$ (13). Stochastic variations in the expression of reporter genes encoding fluorescent proteins are reflected in heterogeneity in the levels of these proteins in individual cells. The work on gene expression noise in eukaryotes (predominantly high-throughput genome-wide studies) has generally emphasized the influence of cell-to-cell variations in mRNA abundance that are driven by fluctuations in transcription, whereby correlations have been identified between noise level and variables that include promoter structure, gene function and chromatin density (16,20).

In contrast, until recently, there has been very little progress in understanding how posttranscriptional steps might contribute to noise generation. One comparable study in the yeast *Saccharomyces cerevisiae* found that noise strength for *GFP* gene expression increased linearly with translation efficiency (varied by changing codon usage; (21). On the other hand, two other studies in yeast indicated that intrinsic noise scales inversely with protein abundance (20,22), but did not differentiate between transcriptional and posttranscriptional contributions. In contrast, intrinsic noise in mammalian cells does not always show this relation at lower protein abundance values (23). Other work suggested that a high tRNA adaptation index is correlated with noise (24). Given the apparent contradictions in previous research, we recently performed detailed studies of the contribution of translational events to gene expression noise generation (25,26). These studies on reporter genes have shown that constraints on translation initiation imposed by structural elements in the 5’UTR provide an additional source of noise. This suggested that more complex endogenous 5’UTRs could impose a variety of noisy behavioural features on the expression of eukaryotic genes.

In this context, it is relevant to consider how gene expression noise can be linked to stochastic switching between states that influence cell viability in variable environmental conditions. This has led to discussion of the concept of ‘bet-hedging’, which is a term borrowed from the financial sector to describe investment in opposite outcomes in order to provide protection against monetary losses. Analogous strategies based on non-genetic heterogeneity (noise) are now thought to underpin viability and/or survival in a number of organisms. For example, slow-growing ‘persister’ cells of *Escherichia coli* can withstand extended exposure to antibiotic treatment, and can switch to faster growth once the antibiotic is removed (27). Human melanomas can contain small subpopulations of cells that divide slowly and are thus resistant to chemotherapy (28). Transcriptional bet-hedging cases have been identified in *S.cerevisiae*: first, slower-growing cells that produce higher levels of the trehalose-synthesis regulator Tsl1 enhance the probability that a population will survive heat stress (29); second, probabilistic activation of the galactose gene regulatory network in a subpopulation of cells enables yeast to undergo a more rapid metabolic transition from glucose to galactose (30). However, understanding of the mechanistic basis of such bet-hedging phenomena is limited and, looking at the wider picture, it is evident that the potential contribution of translation has received very little attention. At the same time, there is evidence that cell-to-cell heterogeneity can also be advantageous in stable, even benign, environments (31).

**Figure 1.**
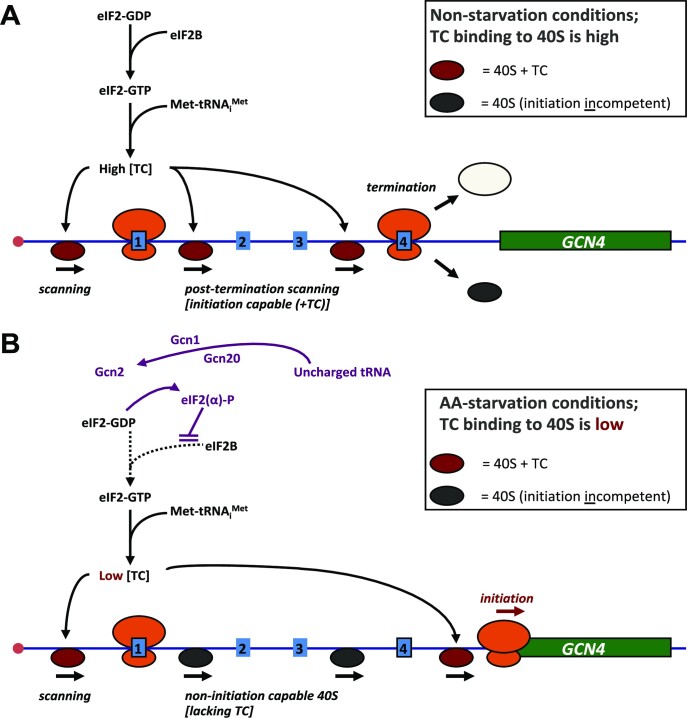
*GCN4* translational regulation scheme. According to this extensively tested model (32), at high amino acid availability (**A**), normal physiological levels of the ternary complex (TC: GTP-eIF2-Met.tRNA_i_^Met^) drive rapid binding to the 40S ribosomal subunit, enabling it to scan effectively. Moreover, TC binds 40S relatively rapidly (post-termination) downstream of uORF1 (an uORF that promotes post-termination scanning and re-initiation), so that the 40S can resume scanning and recognize a downstream uORF (reinitiate). If this downstream element is uORF4, the context of the termination codon promotes release of ribosomes and prevents further scanning. In non-starvation conditions therefore, uORF4 acts as a block to initiation on *GCN4*. When amino acid availability is low (or this state is mimicked by adding the inhibitor 3-AT) (**B**), high levels of uncharged tRNA cause the Gcn2 kinase to phosphorylate eIF2α, which in turn inhibits the GDP-GTP exchange activity of eIF2B. This causes a reduction in the intracellular TC abundance, thus slowing the rate of TC binding to the 40S subunit. Thus, many 40S subunits now scan through uORFs 2–4 without reinitiating, only to bind TC in the long region between uORF4 and the *GCN4* main ORF, *thus enabling (re)initiation on GCN4*. The *GCN4* 5’UTR (5’UTR*^GCN4^*) retains much of its regulatory capacity after removal of uORFs 2 and 3 (32), and therefore we have focused on the roles of uORFs 1 and 4 here. The stochastic properties of 5’UTR*^GCN4^*-mediated translational regulation are described for the first time in the current work.

In this new study, we have turned our attention to the stochasticity of translation initiation on another example of a complex 5’ untranslated region, that of *GCN4* in *Saccharomyces cerevisiae*. An extensive body of previous work has identified the key deterministic features of translational regulation of the *GCN4* transcriptional activator in terms of cell-population data averages (32,33); (Figure 1). The Gcn4 protein upregulates the transcription of genes involved in a large number of biosynthetic pathways, including those for amino acids, purines and vitamin-cofactors, and also activates synthesis of mitochondrial carrier proteins, amino acid transporters and autophagy proteins. Translation via the *GCN4* 5’UTR (5’UTR*^GCN4^*), which contains four short upstream open reading frames (uORFs), is induced in response to starvation of amino acids, purines or glucose, as well as exposure to sodium chloride and rapamycin (32). Under non-starvation conditions, the fourth uORF in the *GCN4* 5’UTR is generally assumed to act as a barrier to translation of the downstream open reading frame corresponding to the *GCN4* coding DNA sequence (CDS) by promoting dissociation of ribosomal subunits and mRNA molecules following termination of its encoded three-amino-acid polypeptide (Figure 1); (32). In contrast, uORF1 functions as a positive element that promotes further downstream scanning (‘rescanning’) that allows rebinding of the ternary complex (TC: GTP-eIF2-Met.tRNA_i_^Met^) and thus reacquisition of the competence to initiate on downstream start codons, most importantly uORF4. uORFs 1 and 4 are known to be the major regulatory elements acting on translation of *GCN4*, since removal of uORFs 2 and 3 has only a minimal effect on the regulatory functionality of the 5’UTR*^GCN4^* (32). Under induction (nutritional stress) conditions, phosphorylation of the α subunit of eIF2 [by general control nonderepressible 2 (Gcn2) kinase] converts eIF2 to a competitive inhibitor of the guanine nucleotide exchange factor eIF2B. This has two effects: first, it suppresses global protein synthesis; second, it modulates the re-initiation kinetics of ribosomal pre-initiation complexes scanning downstream of uORF1 so that some can scan past uORF4 and re-initiate instead on the start codon of the *GCN4* CDS, thus differentially activating translation of this reading frame (Figure 1).

In contrast to the earlier body of *GCN4*-related research mentioned above, the present study focuses on regulatory heterogeneity at the single-cell level. We find that a subset of cells in any given yeast population manifests a high 5’UTR*^GCN4^*-mediated expression state under non-starvation (non-induced) conditions, and we investigate the stochastic processes that underpin this phenomenon. We conclude that the 5’UTR*^GCN4^* generates a previously unknown type of translational stochasticity that, in turn, results in a corresponding degree of cellular heterogeneity with respect to activation by Gcn4 of biosynthetic pathways under non-starvation conditions. We also illustrate, using a newly developed mathematical model, that the existence of the sub-population manifesting enhanced 5’UTR*^GCN4^*-mediated translation can only be explained in terms of stochastic events that are not inherent to the canonical *GCN4* regulatory model that applies to population averages.

## MATERIALS AND METHODS

### Strain construction

The *Saccharomyces cerevisiae* strains used in this study were all derived from the background strain PTC830: MATα ura3-1 leu2-3, 112 his3-11, 15 can1-100 (a derivative of W303). The ymNG expression reporter constructs were integrated at the *CAN1* locus. The genes encoding yeGfp and mRuby3 fluorescent proteins were integrated at the C-termini of the natural genomic *GCN4* cds and *ADE8* cds, respectively.

### Flow cytometry, cell sorting and gene expression stochasticity analysis

Cells were prepared for flow cytometry as described previously (25,26). Yeast cells expressing the yEGFP or ymNeonGreen reporter genes were excited using a 488 nm laser, and fluorescence was collected through 505 nm long-pass and 530/30 nm band-pass filters on a BD Fortessa X20 flow cytometer. For dual-colour reporter strains, yEGFP or ymNeonGreen was excited and fluorescence was collected using the same laser and filters as described above, while mRuby3 was excited using a 561 nm laser and its fluorescence collected through a 600 long-pass plus 610/20 nm band-pass filters. The data were recorded using the ‘Area’ option. Flow cytometry data were exported from the acquisition program (FACSDiva) in the FCS3.0 format with a data resolution of 2^18^. A custom R programme was written [using flowCore, flowViz and flowDensity Bioconductor packages; as described previously (25,26)] to calculate statistics for each file.

For calculating the coefficients of variation, cytometry files were processed as follows: (i) the first second, and final 0.2 s, of data were removed to minimize errors due to unstable sample flow through the cytometer; (ii) thresholds of 40 000–100 000 and 10 000–90 000 for the FSC and SSC gates, respectively, were typically used to limit the influence of cellular debris and aggregated cells; (iii) for the remaining data, the FSC and SSC values of the highest density centre of the FSC–SSC scatterplot were calculated, and the distance of the ith sample to the centre was determined: distance *i* = √((FSC *i* – FSC centre)^2^ + (SSC *i* – SSC centre)^2^); (iv) the fluorescent reporter (e.g. ymNG) data within the radius were used to calculate the coefficient of variation (CV), i.e. CV = σ/μ. The intrinsic, extrinsic and total noise from dual-color reporter flow cytometry data were calculated as described previously (25,26).

Fluorescent reporter (e.g. ymNG) data were obtained from six independent experiments, whereby the centre point for the scatter plot analysis was either set automatically, or manually at FSC = 59 000/SSC = 27 000. The average number of cells analyzed given a radius limit of 4000 was approximately 780 (900). This gate radius was chosen as a compromise point at which, over multiple experiments, the variation between experiments was minimal and the number of cells analysed provided statistically meaningful results. This procedure is similar to one reported previously (20) except that, by focusing on the cell density centre, we have been able to maximize the number of cells that are sampled.

### Live-cell imaging and data analysis

One day prior to an experiment, single colonies from each of the strains were picked and grown overnight in YNB (plus amino acids; 2% glucose) to saturation with shaking at 30°C. The following morning, cells were diluted to give an optical density at 600nm (OD_600_) of ∼0.2, and these diluted cultures were grown to mid-log phase (OD_600_ ∼0.6) with shaking at 30°C. This procedure allowed us to maintain the cultures in the exponential growth phase right up to the time of measurement. 3 μl of mid-log phase culture from each strain were loaded onto clean slides for imaging acquisition.

Live-cell images were acquired on a Nikon Ti Spinning Disk confocal microscope, equipped with a 60× objective, 1.4 numerical aperture using 1.515 refractive index oil and an ANDOR DU-888X EMCCD camera. The temperature of the incubation chamber was set at 30°C. Cells were imaged in two dimensions. Images were acquired for each field of view using yEGFP/ymNeonGreen (excitation 488 nm, emission 521 nm) and mRuby3 (excitation 561 nm, emission 610 nm) filter sets in this order. The background strain PTC830 (not expressing any reporter gene) was imaged alongside the sample strain as a negative control.

Cell segmentation and image analysis were performed using a custom-written R programme (using EBImage Bioconductor packages). A common pre-processing step involves cleaning up the images by removing local artifacts or noise through smoothing. Images were smoothed using the wrapper function which performs Gaussian filter smoothing. Image segmentation was performed to identify the individual cells using the watershed algorithm over the binary image (34).

### CRISPR/cas9 genome editing

In this study, the site‐directed mutant strains were generated using CRISPR-Cas9 genome editing technology as described previously (35). The vector (pML104) for the one-plasmid system of CRISPR-Cas9 gene editing (www.addgene.org) contains both the Cas9 gene and the guide RNA expression cassette. To design the specific single guide RNA (sgRNA), we used an online tool (http://wyrickbioinfo2.smb.wsu.edu/crispr.html) to aid identification of unique guide RNA target sites in the yeast genome. RNA expression cassette double-stranded DNA fragments (gBlocks) corresponding to the selected guide RNAs were synthesized by Integrated DNA Technologies. Yeast transformations for CRISPR-Cas9 gene editing were performed as described previously (35). A synthetic double-stranded gBlock template with the targeted gene mutation was used to introduce each nucleotide substitution. To validate creation of the mutants, transformants were isolated for genomic DNA extraction and sequence analysis.

### Microfluidic devices for single cell studies

The configuration of the microfluidic devices used in this work (Supplementary Figure S1) was based on the principle of hydrodynamic pressure holding individual cells within suitably designed jail traps comprising PDMS pillars. We derived our devices from a design outlined previously (36), and used a chrome-plated glass photolithography mask for their manufacture at a tolerance of ±0.15 μm. After calibration of the spreading of the SU8 photoresist by a spin coater, silicon wafer moulds were created to the desired dimensions and tolerances. These moulds were utilized in the manufacture of PDMS casts for use in the microfluidics experiments. Media flow through the assembled microfluidics devices was managed using a Fluigent pressure-controlled system, which allowed automatic feedback under the control of Fluigent AIO software. The statistics of individual cell division rates during experiments confirmed that nutrient provision in the microfluidics device supported exponential growth at rates (cell-cycle completion time 88 ± 21 min) comparable with the maximal rates observed in the low-density exponentially growing batch cultures used elsewhere in this work. Cell clumps were removed from cell cultures prior to loading into the microfluidics device using a combination of mild sonication (15 s at low power) in a sonication bath and filtration through a 10 μm filter. Control experiments using flow cytometry and live-cell imaging showed that short exposure to such low power sonication elicited no detectable stress response from the cells.

### Computational modeling

The model is derived from the totally asymmetric simple exclusion process [TASEP; (37)], as applied previously to *GCN4* translation (38). Full details are presented in the *Supplementary Data* section. Additional features were introduced that enable the new model to provide an explanation of the SET*^GCN4^* phenomenon, as outlined in the Results section.

## RESULTS

### Heterogeneity of expression from the *GCN4* 5’untranslated region

**Figure 2.**
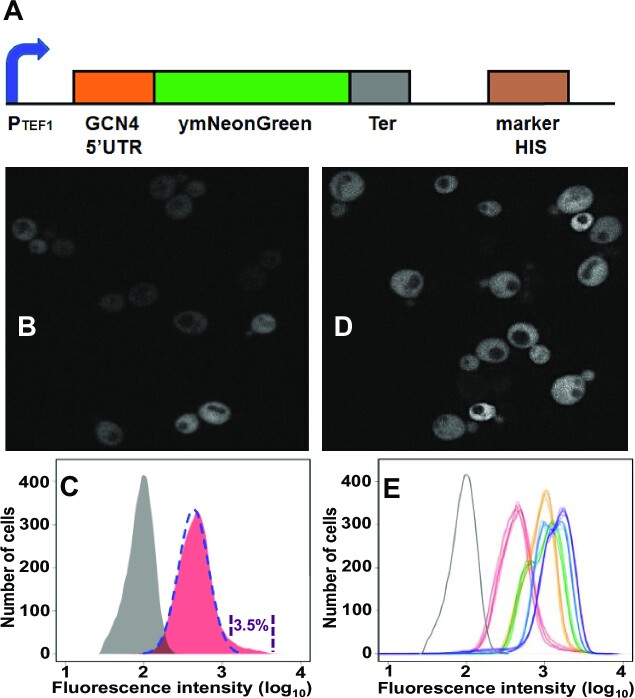
Stochastically Enhanced Translation state (SET*^GCN4^*) cells. (**A**) A genomic ymNeonGreen reporter construct including a constitutive promoter (P*_TEF1_*) and the 5’UTR*^GCN4^* was established in *S.cerevisiae*. Fluorescence microscopy (**B**) and flow cytometry (**C**) revealed the presence of a small sub-population (approximately 3.0%) of SET*^GCN4^* cells that are in a high-expression state despite non-starvation conditions (shown here to lie outside of the normal distribution of fluorescence intensities, indicated by the broken line). The fluorescence distribution of cells carrying the genomic reporter construct is shown in red and the (auto-) fluorescence distribution of a control strain lacking the genomic integration is shown in grey (**C**). The fluorescence intensity distribution of the SET*^GCN4^* cells maps onto that assumed by the total population of cells after 4 h of 3-AT induction (purple curve in panel E). (**E**) For comparison, 3-AT induction resulted in the progressive transition of all cells of the reporter strain from a low state of fluorescence to a high state. The flow cytometry distributions [only the outline of each distribution (six biological repeats for each time point) is shown] are colour-coded according to the period of induction: 0 time (red), 1 h (yellow), 2 h (green), 3 h (blue) and 4 h (purple). (**D**) Fluorescence microscopy confirms that all of the cells manifest the high-fluorescence state after 4 h of 3-AT induction (purple distribution in panel E). The cell fluorescence in panels B and D is represented in white to facilitate identification of different intensities of reporter activity.

We investigated the expression characteristics of the *GCN4* system at the single-cell level using a genomic fusion construct comprising the *GCN4* 5’UTR (5’UTR*^GCN4^*) coupled to the yeast-optimised mNeonGreen coding sequence (ymNG CDS; Figure 2A). In order to focus on translational control mediated by the 5’UTR*^GCN4^*, this construct was placed downstream of the constitutive P*_TEF1_* promoter. We reported previously that this promoter generates single cell mRNA copy number data that fit well to a standard negative binomial distribution across a yeast population (26). Examination of the cell-to-cell heterogeneity of ymNG expression with the help of fluorescence microscopy revealed that the cell population did not uniformly manifest the non-induced state (Figure 2B). Unexpectedly, flow cytometry revealed that a significant proportion (≥3.0% of total cells) of the population manifests high fluorescence intensity (Figure 2C), while most of the cells show lower fluorescence activity that is consistent with the expected non-induced state. Indeed, the intensity range recorded for the stochastically enhanced translation state (SET*^GCN4^*) sub-population of cells mapped to the fluorescence distribution observed for cells responding to amino-acid starvation, as induced by the addition of 3-aminotriazole (3-AT; Figure 2D, E), a competitive inhibitor of imidazoleglycerol-phosphate dehydratase (encoded by *HIS3*) whose addition mimics amino acid starvation (32). In summary, the SET*^GCN4^* cells seem to conform to a separate distribution from the main fluorescence distribution of non-induced cells, whereby the SET*^GCN4^* distribution is narrower, has a higher fluorescence intensity mean, and accommodates approximately 3% of the total population. As we consider in more detail later in this paper, the fluorescence intensity data for the whole population do not fit to a single normal or skewed-normal distribution. A full analysis is provided in the *Supplementary Data* section (including the embedded Supplementary Figure S10 in that section).

### A stochastic asymmetric expression distribution determined by the *GCN4* 5’UTR

**Figure 3.**
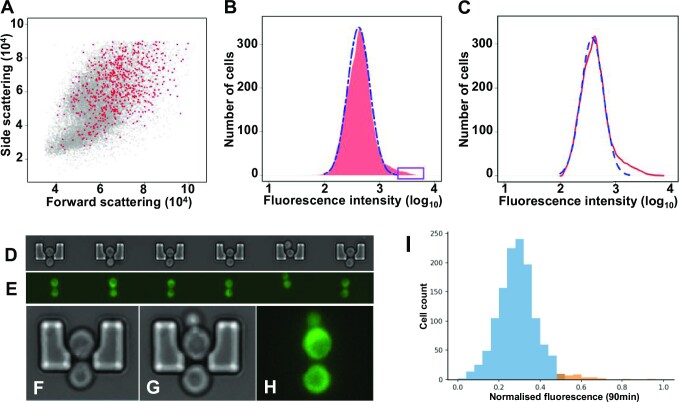
The SET*^GCN4^* state is generated stochastically. (**A**) A plot of forward-scattering vs side-scattering data for the reporter construct strain (described in Figure 2A) reveals that the SET*^GCN4^* state is observed in cells (highlighted in red) with a wide variety of sizes, shapes and internal structures. (**B**) The cells manifesting fluorescence intensity greater than the 99th percentile value (boxed region at upper end of the intensity distribution) were separated using a fluorescence-activated cell sorter (FACS). The blue dotted line represents the predicted outline of a normal distribution of fluorescence intensity. The cells from the boxed region (representing in this case the top 1% of the fluorescence intensity distribution) isolated in this way all manifested the SET*^GCN4^* state, but upon continued growth re-established the original fluorescence intensity distribution, as demonstrated by further fluorescence cytometry analysis (**C**). Cells were also trapped in a microfluidics device and imaged using time-lapse bright field (**D**) and time-lapse fluorescence (**E**) microscopy. The occupied cell traps selected for inclusion in panel E all contain typical dividing SET*^GCN4^* cells, thereby illustrating active cell division in the microfluidic system. Other images show individual trapped cells progressing through the cell cycle and shedding buds through the trap exit door (**F–H**). The respective cells shown here are held in the traps by the pressure of the unidirectional flow of growth medium. An example of one sample population studied in the microfluidics system (*n* = 1269 cells; panel **I**) illustrates the consistently observed result that the most highly fluorescing 3% (orange) belong to a subset (corresponding to SET*^GCN4^*cells) that is distinct from the normal distribution of the other 97% (blue). The smaller number of cells analysed in this way leads to coarser granularity in the fluorescence intensity distribution (I) compared to the flow cytometry data (B).

The observation that, in an exponentially growing culture, there is a sub-population of non-starved cells in which expression of the CDS downstream of the 5’UTR*^GCN4^* is at a level characteristic of starvation-stressed cells led us to investigate in further detail the noise characteristics of translation mediated via this 5’UTR (as manifested in cell-to-cell heterogeneity). A plot of forward-scattering vs side-scattering data (Figure 3A) for the reporter construct strain (described in Figure 2A), plus a version of this plot highlighting the distributions of these variables for the respective cell populations (Supplementary Figure S2), reveal that the SET*^GCN4^* state is observed in cells (highlighted in red) with a wide variety of sizes, shapes and internal structures. We used fluorescence-activated cell sorting (FACS) to isolate cells belonging to the most intensively fluorescing half of the SET*^GCN4^* sub-population (Figure 3B) from the remaining cell population and asked the question whether this sub-group would maintain its high-expression status over further generations of growth. The result was striking: within 10 generations (<20 h) of further growth, the original dominant non-induced fluorescence distribution overlapping with the smaller SET*^GCN4^* sub-population was re-established (Figure 3C). The same result was achieved if solely non-SET*^GCN4^* cells were selected as the starting point for this regrowth experiment. In other words, the asymmetric expression distribution (including the SET*^GCN4^* state) is a default status that is determined by stochastic processes that are inherent to any randomly selected population of yeast cells. Therefore, the SET*^GCN4^* sub-population does not correspond to a distinct genetic variant.

Each flow cytometry experiment reveals the distribution of fluorescence intensity in the individual members of a cell population within a relatively short time window. We therefore utilized microfluidics in combination with time-lapse fluorescence microscopy to perform continuous live-imaging of exponentially growing individual cells within a cell population over the full cycle of cell division. Small volumes of exponentially growing yeast cultures were introduced into a temperature-controlled microfluidic device so that the trap array was populated with single (budding) cells (illustrated by images of selected cells in Figure 3D–H). The growth environment within the microfluidics device was maintained in a stable steady-state using precisely regulated pressure-driven pumps that continuously refreshed the growth medium. The fluorescence intensity of cells that remained immobilized (while still growing normally) in the microfluidic traps was observed over 90 minutes, revealing a distribution of fluorescence values across the trapped population (see an example of one such experiment in Figure 3I). Monitoring more than 1000 single growing cells over an extended timeframe in this way again revealed the existence of the highly fluorescing SET*^GCN4^* sub-population, whereby the relatively small cell sample size (compared to the flow cytometry experiments) resulted in a coarser distribution, as well as more variability between experiments. The latter is highlighted here by the orange-coloured bars corresponding to the cells manifesting the top-3% fluorescence intensities (see the example experiment shown in Figure 3I; the bin size affects the granularity of the graph). In conclusion, this individual-cell-imaging based analysis confirms the existence of the SET*^GCN4^* sub-population as well as its main quantitative features.

### *GCN4* 5’UTR structure underpins the SET phenomenon

**Figure 4.**
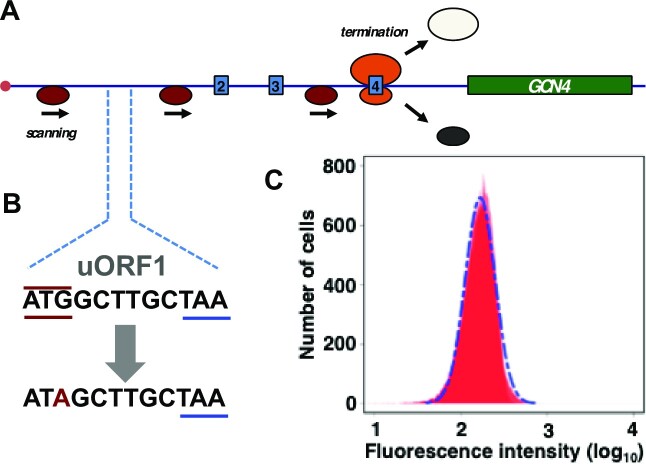
Mutation of the uORF1 start codon to ATA abrogates the SET*^GCN4^* state. (**A**) The uORF1 ATG start codon in the genomic reporter construct (see Figure 2A) was mutated to ATA (**B**). Flow cytometry (of exponentially growing, non-starved cells) revealed that the resulting strain no longer supports generation of the SET*^GCN4^* state (**C**).

In further experiments, we investigated the role of the 5’UTR*^GCN4^* in generating this stochastic distribution. An important feature of the 5’UTR*^GCN4^* is the influence of its uORFs on the behaviour of ribosomal complexes that interact with it. Elimination of uORF1 has previously been shown to abrogate translational derepression mediated by the 5’UTR*^GCN4^*; the explanation for this loss of regulation is that the modification allows a greatly increased percentage of ribosomal pre-initiation complexes to initiate on uORF4 and then to dissociate from the mRNA, so that very few of them can initiate on the *GCN4* CDS (32). We found that mutation of the uORF1 start codon to AUA markedly reduced the size of the SET*^GCN4^* sub-population (Figure 4). It also changed the reporter expression behaviour brought about by 3-AT induction, yielding an overlapping bimodal fluorescence distribution and, overall, a threefold-reduced mean value for the reporter fluorescence (Supplementary Figure S3). This outcome is consistent with the primary role of uORF1 as a reinitiation-promoting structural element whose functional impact can only be partially reproduced by uORF2, at least when located in its normal position within the 5’UTR*^GCN4^* (32). In further experiments, we replaced uORF1 by uORF4. In line with the demonstrated property of uORF4 to promote ribosomal release after termination on its stop codon, this modification also markedly limited the induced level of gene expression and strongly reduced the size of the SET*^GCN4^* sub-population (data not shown). These were very similar outcomes to those observed after modification of the uORF1 start codon to AUA (Figure 4 and Supplementary Figure S3). We conclude from this section of the work that modifications of the uORF-based architecture of the 5’UTR*^GCN4^* that partially or fully eliminate the nutrient-stress-induced translation regulatory mechanism also suppress development of the SET*^GCN4^*state. The fluorescence distributions observed in these cases are similar to those manifested by a control reporter construct featuring a short unstructured 5’UTR that does not contain uORFs, as considered in the modeling discussion in the *Supplementary Data* section (specifically the embedded Supplementary Figure S12 in that section).

### The role of eIF2α phosphorylation

**Figure 5.**
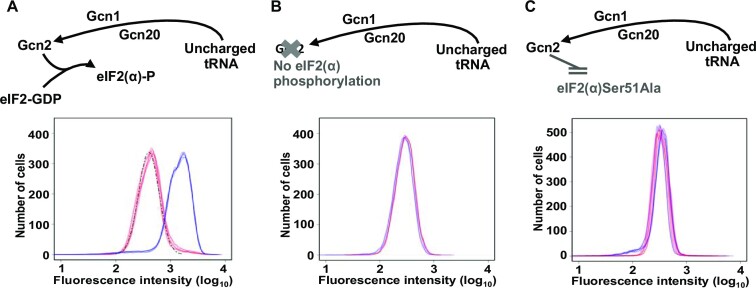
Blocking phosphorylation of eIF2α eliminates the SET*^GCN4^* state. Flow cytometry analysis of genomic reporter gene expression (Figure 2A) in strains lacking Gcn2 kinase [*gcn2Δ*; (**B**)] or with a Ser51Ala mutation in eIF2α (**C**) reveals the loss of the SET*^GCN4^* sub-population [compare wild-type control (**A**)]. In each case, cells that had been growing exponentially in YNB medium were subjected to flow cytometry. The fluorescence profiles for cells growing in YNB medium alone (red lines) are overlaid with profiles of cells in YNB plus 3-AT (4 h of induction; blue lines). For the purpose of clarity, the flow cytometry data from six biological repeats of each experiment are represented by the (overlaid) outlines of the respective distributions.

The degree of phosphorylation of eIF2α is known to play a key role in translational regulation of *GCN4*. It modulates the ability of reinitiating ribosomal 43S complexes to bypass uORF4 and thus to initiate translation on the start codon of the main *GCN4* CDS (32). We tested the hypothesis that the existence of the SET*^GCN4^* cells is linked to eIF2α phosphorylation. We did this in two ways. First, we compared the distribution of fluorescent reporter activity in a *gcn2Δ* genetic background (Figure 5B) with that obtained with a wild-type control strain (Figure 5A; see also overlay of these respective panels in Supplementary Figure S4). Flow cytometry revealed that, in the absence of Gcn2 kinase, translational activation of the genomic *GCN4* reporter construct in the wider population was, as expected from earlier work (32), abrogated. In addition, we observed that the SET*^GCN4^* cells were no longer evident (Figure 5B). Second, we observed a very similar result using a strain in which the phosphorylation target of Gcn2 kinase, eIF2α Ser51, was mutated to Ala (Figure 5C). In the latter case, activation of Gcn2 (through addition of 3-AT) in the eIF2α Ser51Ala mutant cells leads to a small degree of narrowing of the expression profiles of the reporter construct in the non-activated and activated states (Figure 5C), perhaps because of some other effect of Gcn2 activation in the cell. Overall, the most consistent feature of both types of experiment was the disappearance of the SET*^GCN4^* sub-population (compare, for example, the profile delineated by the broken vertical lines in Figure 2C).

### Extrinsic versus intrinsic noise

**Figure 6.**
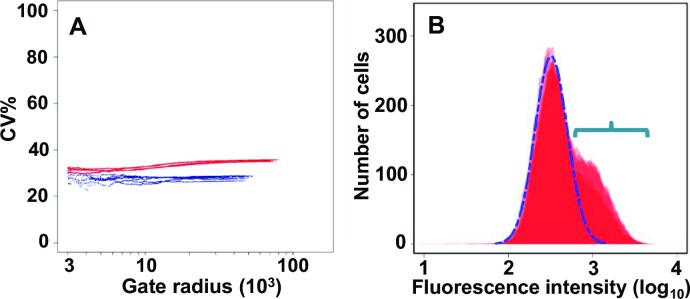
Gating analysis of up-regulated expression dependent on the *GCN4* 5’UTR. (**A**) Light-scatter gating analysis^20^ indicates that expression dependent on the 5’UTR*^GCN4^* manifests a comparatively high level of noise (red data points), even in the absence of induction by starvation (or 3-AT). The plot reveals the effect of progressively constraining the heterogeneity of the cells (by reducing the gate radius) on the %CV (coefficient of variance as a percentage) value. As the gate radius is constrained (moving along the x-axis from right to left), the contribution of extrinsic factors is reduced, and ultimately a minimum value is reached that is primarily attributable to the intrinsic noise component. The SET*^GCN4^* sub-population of cells (blue data points, representing those cells whose ymNG fluorescence intensity lies above the 97th percentile of total population fluorescence intensity values) manifests a lower level of overall noise, indicating that there is greater homogeneity in the rates of *GCN4* gene expression in these cells, and a smaller extrinsic component. (**B**) Flow cytometry fluorescence intensity profile of a strain carrying the genomic P*_TEF1_*-5’UTR*^GCN4^*-*ymNG* construct (see Figure 2A) plus a plasmid-borne *GCN2* gene that overproduces the Gcn2 protein (to stochastically varying degrees because of copy number variations). We observed a high-noise, (overlapping) bimodal distribution that features peaks typical of non-induced cells (major peak outlined by broken blue line) and of a greatly increased proportion of SET*^GCN4^* cells (see region indicated by the horizontal blue bracket).

In order to obtain more information about the translational stochasticity generated by the 5’UTR*^GCN4^*, we used a previously reported procedure to apply light-scatter gating analysis to the fluorescence cytometry data (20,26). This procedure is generally used to provide information on the relative contributions of extrinsic and intrinsic components to the total gene expression noise observed in a given population of cells. We applied this procedure to the population of uninduced cells of a strain carrying the chromosomally integrated reporter construct shown in Figure 2A. The light-scatter gating analysis reveals an interesting property of the SET*^GCN4^* subpopulation of cells (Figure 6A); in comparison with the whole population (red plots), translation initiation mediated by the 5’UTR*^GCN4^* in the SET*^GCN4^* cells manifests a much reduced component of extrinsic noise (blue plots). Assessment of the smaller gate radius data also indicates that the SET*^GCN4^* subpopulation manifests a lower intrinsic noise level (blue plot lines in Figure 6A). These observed changes in noise characteristics can be reproduced by the system model we describe below.

Since we had already demonstrated that the presence of an active Gcn2 kinase is linked to the expression state in SET*^GCN4^* cells (Figure 5), we examined whether variation in the basal activity of this kinase might potentially constitute one form of extrinsic noise that could contribute to the generation of the bimodal distribution of 5’UTR*^GCN4^*-supported translation initiation that is observed in non-induced cell populations. We transformed the P*_TEF1_*-5’UTR*^GCN4^*-*ymNG* strain using a centromeric plasmid carrying *GCN2*. Since centromeric plasmids can manifest mean copy numbers per cell of up to at least five (39), expression of a promoter-gene combination from such a plasmid can generally be expected to be increased, and show enhanced variability compared to the same promoter-gene combination in a genomic locus. The presence of a significantly increased basal activity of Gcn2 kinase will, even in the absence of induction (by starvation or the addition of 3-AT), result in an increased level of phosphorylation of eIF2α. The results of the flow cytometry experiments (Figure 6B) reveal that this markedly increases the size of the sub-population of SET*^GCN4^* cells, consistent with the proposal that variations in Gcn2 activity represent a natural generator of extrinsic noise in the 5’UTR-mediated regulatory system controlling *GCN4* translation.

### Expression state of *gcn4*-activated biosynthetic pathway genes

**Figure 7.**
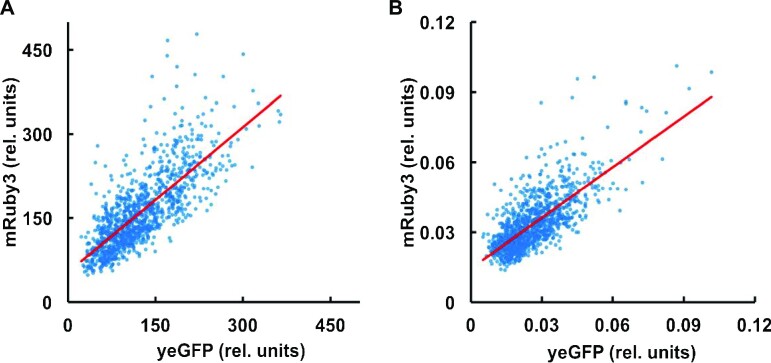
Enhanced *ADE8* expression in SET*^GCN4^* cells. A strain was studied that carried genomic integrations of both of the gene fusions *ADE8::mRuby3* and *GCN4::yEGFP*. Fluorescence microscopy of this strain growing in YNB medium revealed the *ADE8::mRuby3* expression level in each cell in the studied population, and the measured fluorescence value was compared with the fluorescence generated by *GCN4::yEGFP* expression in the same cell. Plotting this pair of values for the whole population reveals a strong correlation between *ADE8::mRuby3* fusion reporter intensity and *GCN4::yEGFP* fusion reporter construct intensity in individual cells, indicating that, in SET*^GCN4^* cells, the non-stress-induced (but stochastically enhanced) level of *GCN4::yEGFP* correlates with a correspondingly higher level of transcription of *ADE8::mRuby3*. Towards the higher end of the non-induced level of intracellular Gcn4::yEGFP abundance in the distribution, the Ade8::mRuby3 abundance in each cell skews more positive relative to the linear correlation line (red). Panel **A** shows the plot of total fluorescence intensity per cell for the respective reporters, while the values in panel **B** are normalised to (divided by) the volume of each cell.

If the SET*^GCN4^* state is linked to an increased level of the Gcn4 transcriptional activator in these cells, this should switch on expression of downstream genes involved in amino acid biosynthesis. We accordingly integrated *yEGFP* at the C-terminal end of the genomic *GCN4* CDS and mRuby3 at the C-terminal end of the genomic *ADE8* CDS. *ADE8* is a Gcn4-regulated gene that encodes phosphoribosyl-glycinamide transformylase, an enzyme that catalyses a step in the purine nucleotide biosynthetic pathway. This enabled us to measure both the relative *in vivo* abundance of the *Gcn4*::yEGFP fusion and, in parallel, the degree of downstream transcriptional activation imposed by this fusion protein in exponentially growing cells. Analysis of the fluorescence characteristics of randomly selected, non-induced, individual cells revealed the relationship between the intracellular abundance of the yEGFP fusion protein and *ADE8::mRuby3* expression. We identified a strong correlation between the abundance of the respective fusion proteins (Figure 7). It is also noticeable that a large percentage of those cells manifesting the highest yeGFP fluorescence values (including the SET*^GCN4^* sub-population) also manifest above-proportional increases in mRuby3 fluorescence. This is consistent with increased activation of the P*_ADE8_* promoter at higher levels of Gcn4. Overall, these data confirm that in all cells of the population, including the SET*^GCN4^* cells, *ADE8::ymRuby3* was subject to *GCN4::yEGFP*-dependent induction.

### A possible mechanism underpinning the SET*^GCN4^* state

**Figure 8.**
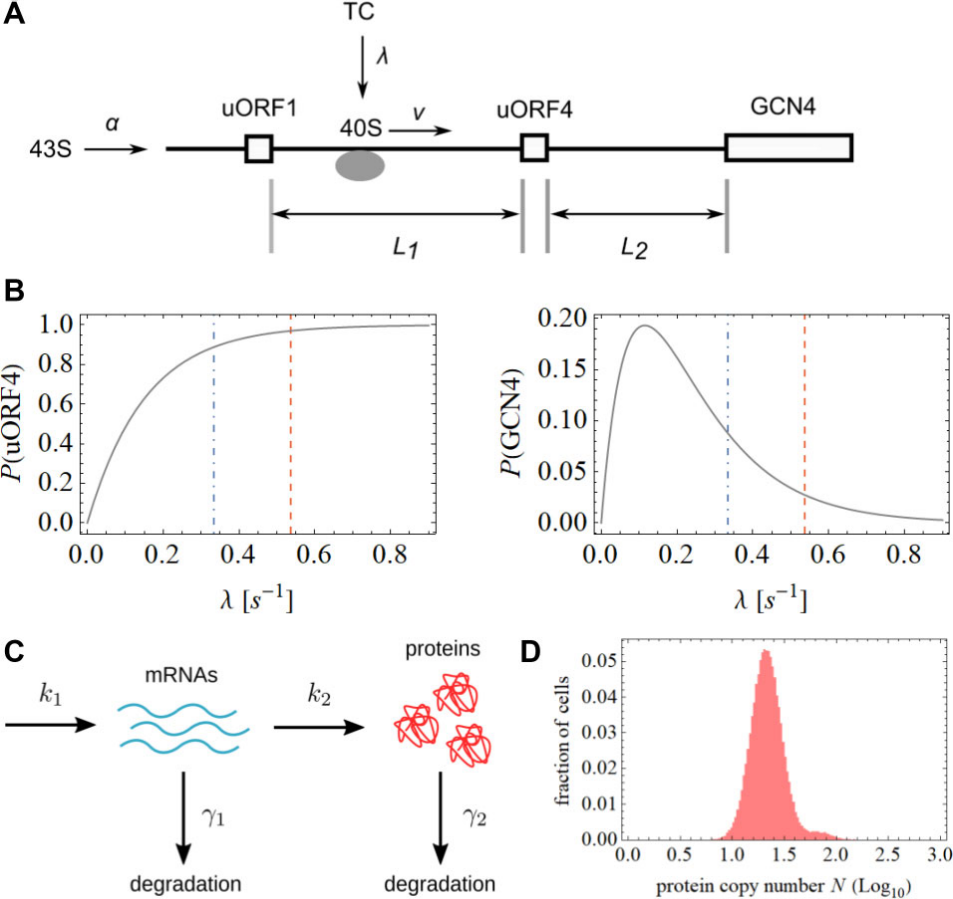
Model of stochastic events on the 5’UTR*^GCN4^*. A number of variables were incorporated into the computational model (**A**). This model predicts relationships between the rate of TC binding to scanning 40S and the rate of reinitiation on uORF4 and on the *GCN4* coding region, respectively (**B**). The main distribution of protein synthesis (in each cell) is dictated by a number of processes that occur simultaneously (**C**), and can be explained assuming the value of the translation rate under repressing conditions. In contrast, the SET*^GCN4^* cell distribution can be explained by cell-to-cell variations in the TC binding rate. Here, we have modeled the generation of the TC binding rate variations in response to variations in Gcn2 activity. Combining these two strands of modeling, we obtain an asymmetric profile that is remarkably close to the observed behavior of the *GCN4* system (**D**).

We explored whether we can model a scenario in which a population of cells manifests a variation in an activator that provides an explanation for the observed abundance of SET*^GCN4^* cells. Understanding what assumptions are required to simulate the results that we have observed helps us to elucidate the likely mechanistic basis of the SET*^GCN4^* phenomenon. We have applied a model of *GCN4* translation (Figure 8A) that is based on the totally asymmetric simple exclusion process (TASEP; 37) as more recently applied to *GCN4* translation (38). The model involves binding of the 43S complex to the 5’ end of mRNA at rate *α*, followed by scanning at rate *v* and initiation at uORF1. Following termination of uORF1 translation, the small ribosomal unit (40S) remains on the transcript with a probability *η* and scans at rate *v* to the next start codon, which is located at the start of either uORF4 or the *GCN4* CDS. We have ignored uORF2 and uORF3 in this version of the model because the regulatory properties of the *GCN4* 5’UTR are largely maintained in their absence (32). Downstream of uORF1, a new ternary complex (TC) can bind to the scanning 40S at rate *λ*, forming the complex we refer to as 40S-TC. The model and its parameters are presented and discussed in full detail in the *Supplementary Data* section.

In this model, the rate at which 40S-TC initiates at the *GCN4* start codon is equal to the scanning speed *v* multiplied by the probability that 40S-TC will reach the *GCN4* start codon. We make the assumption that the rate *α* at which the 43S complex binds to the 5’end is much lower than the scanning rate *v*, i.e. that the total density of initiation complexes concurrently scanning the 5’UTR is also low. This assumption is justified by the results of ribosome density mapping experiments that detect the presence on the 5’UTR of only approximately one ribosome under repressing conditions, and approximately two ribosomes under derepressing conditions (40). We have estimated the values of *λ/v* and *η* under repressing conditions (0.018 and 0.62, respectively) on the basis of previous studies; one examining the effects of the respective uORFs on translation events on the *GCN4* 5’UTR (41) and another looking at the dynamics of yeast ribosomal scanning (42). Taking into account the induction behaviour observed using flow cytometry (see, for example, Figure 2E), *λ/v* becomes 0.011 under derepressing conditions whereby, *λ* assumes the value 0.33 s^−1^. Under repressing conditions, *λ* = 0.54 s^−1^. Using a value of 0.08 s^−1^ for *α*, the model predicts the relationships between the value of *λ* and the probabilities of reinitiation on uORF4 and on *GCN4* shown in Figure 8B (see also the *Supplementary Data* section). These plots highlight the respective reinitiation probabilities predicted at the different values of *λ* under repressing and derepressing conditions. The variables in this model can be adjusted to obtain an optimal model fit for a range of experimental conditions.

Building on the above model, we propose a simple rationalization (outlined in detail in the *Supplementary Data* section) of the 5’UTR*^GCN4^*–dependent translation profile observed in a population of yeast cells (as reflected, for example, in the fluorescence data shown in Figure 3B) that comprises a normal distribution of fluorescent reporter expression that overlaps with the enhanced fluorescence distribution corresponding to the SET*^GCN4^* state. We have employed a simple two-step gene expression model to explain these two distributions (Figure 8C). The main normal distribution can be explained assuming the value of the translation rate under repressing conditions. In contrast, the SET*^GCN4^* cell distribution can be explained by cell-to-cell variations in the TC binding rate *λ*. We have demonstrated that one potential cause for these variations is cell-to-cell heterogeneity in the activity of Gcn2 kinase. We expect that *λ* assumes a maximum value of *λ*_repressed_ under conditions in which Gcn2 activity is below a threshold value (see above), while *λ* assumes a lower (derepressing) value at Gcn2 kinase activities greater than the threshold value. We have shown that positive fluctuations of Gcn2 activity can increase derepression in the cell population (see Figure 6). However, negative fluctuations of Gcn2 kinase cannot increase *λ* beyond *λ*_repressed_ because this is limited by the rate control coefficients of other translation initiation factors (43). These considerations generate an asymmetric profile (Figure 8D) that closely resembles the experimentally observed behaviour of the system (Figure 3).

## DISCUSSION

In this study, we have identified a form of non-genetic variation that operates at the translation level. In an exponentially growing yeast culture, this manifests itself in the form of a subset of cells in which the 5’UTR-mediated derepression of *GCN4* expression is activated as the result of stochastic, rather than deterministic, factors. This phenomenon is reproducibly observed under three different sets of experimental conditions that we have studied using flow cytometry, fluorescence microscopy of cells grown in batch culture, and fluorescence microscopy of cells over a longer timescale within a microfluidics environment in which the growth medium is continuously refreshed. The SET*^GCN4^* subpopulation comprises <5% of the total cell population, which is comparable to the limited sizes of subpopulations manifesting transcriptional heterogeneity in relation to other regulatory responses in yeast [for example, carbon source switching (30); stress protection by Tsl1 (29)]. We have investigated the provenance of this state of stochastic enhanced translation of the *GCN4* gene (the SET*^GCN4^* state). The experimental data reported in this study reveal that the SET*^GCN4^* state is underpinned by the operational features of the *GCN4* 5’UTR acting at the translation step of gene expression. Mutational analysis of uORFs 1 and 4 show that these structural elements play key roles in the generation of the SET*^GCN4^*sub-population. This is consistent with the idea that stochastic variation in the mechanisms underpinning the fate (uninterrupted scanning or reinitiation) of pre-initiation complexes as they encounter the uORF4 start codon drives the SET*^GCN4^* phenomenon. We have also demonstrated that the SET*^GCN4^*state is dependent on phosphorylation of eIF2α. Mutational disruption of both uORF-mediated scanning modulation and *GCN2* phosphorylation eliminates the SET*^GCN4^* state and concomitantly reconfigures the fluorescence intensity distribution to a more symmetrical form. Moreover, it is notable that, in those cases (Figures 4 and 5) in which we have inactivated the *GCN4* translational regulation mechanism via previously described mutations (32), the fluorescence intensity data assume the form of a normal distribution (see also the Supplementary Data section). This suggests that, under standard conditions, the mRNA-scanning stochasticity that generates the SET*^GCN4^*state is superimposed on the transcriptional noise driven by the promoter.

At first sight, it is tempting to conclude that the SET*^GCN4^* state is comparable to the phenotype of cells carrying a Gcd^−^ (constitutive derepression) mutation (32). However, our results indicate that the SET*^GCN4^* state is limited to only a subset of cells by virtue of stochastic variation; i.e. it is not generated by a nutritional starvation response. It seems likely that the SET*^GCN4^* state is only feasible if translation of most, if not all, of the *GCN4* mRNA molecules in any given cell is translationally activated, since otherwise the observed high level of expression in each SET*^GCN4^* cell would not be possible. This suggests the involvement of some form of extrinsic noise, i.e. variations in the global cellular environment. The dominant contribution of an extrinsic noise source, such as Gcn2, would also explain the occurrence of a sub-population of individual cells in which *GCN4* translation is evidently activated under non-starvation conditions. Moreover, in another relevant study, translation complex profiling has provided additional insight into the interactions between 40S ribosomal small subunits (SSUs) and *GCN4* mRNA molecules within the cell (44). In that work, translation complex profile sequencing (TCP-seq) has revealed the presence of an SSU footprint over the *GCN4* main ORF AUG at a frequency that suggests that there is a low level of *GCN4* translation on mRNAs extracted from non-starved (non-derepressed) yeast cultures. This TCP-seq result is consistent with the occurrence of the SET*^GCN4^* state in a sub-population of non-starved yeast cells, as described in the present work.

Consideration of the above findings prompted us to explore how cell-to-cell heterogeneity with respect to at least one extrinsic factor might act upon these operational features to generate the SET*^GCN4^* state. Our results indicate that the activity of Gcn2 kinase exerts a strong influence on the SET*^GCN4^* phenomenon. Previous work has demonstrated that inactivation of Gcn2 kinase prevents *GCN4* derepression in response to starvation of yeast cells for amino acids or nucleosides (32). In this study, we have observed not only that inactivation of *GCN2* eliminates the SET*^GCN4^* sub-population, but also that the presence of an excess of Gcn2 activity increases the size of the the SET*^GCN4^* sub-population. These data are consistent with a scenario in which SET*^GCN4^* cells distinguish themselves from the bulk of a yeast cell population by virtue of the exceptionally high level of Gcn2 kinase activity that they possess. This could potentially be due to an enhanced abundance of Gcn2 kinase that is in its basal activity state, since activation linked to induction mediated by the usual nutritional stress pathway would normally be expected to cause marked growth limitation of the SET*^GCN4^* cells, a feature of this sub-population that we have not observed. However, at this stage we cannot rule out the possibility that Gcn2 kinase is subject to partial activation in these cells. For example, partial dephosphorylation of Ser577 in Gcn2, which is known to activate Gcn2-mediated phosphorylation of eIF2α (32), might potentially also be involved, and clarification of this question will need to be the subject of future work. It is notable that 5’UTR*^GCN4^*-mediated noise is reduced in SET*^GCN4^* cells, primarily because of suppression of the extrinsic component. This is consistent with an increased activity of the extrinsic factor that is responsible for generating the SET*^GCN4^* state, although it does not inform us how the increased activity is achieved.

Our observations identify Gcn2 kinase as a source of 5’UTR*^GCN4^*-mediated noise that contributes to the SET*^GCN4^* phenomenon. We have not established whether other factors are also relevant. For example, the actin-binding yeast impact homologue 1 (Yih1) competes with Gcn1 for binding to Gcn2, thus inhibiting the stimulation of Gcn2 kinase by uncharged tRNAs under nutritional stress conditions (45). Future work will therefore need to investigate whether Yih1, or other potential modulators of Gcn2 kinase activity, also play a role in determining the characteristics of the SET*^GCN4^* state. Moreover, it remains to be ascertained whether there exists an alternative route to modulating ribosomal interactions with the 5’UTR*^GCN4^* that is at least partially uncoupled from regulation of global protein synthesis.

Of further note is that there is no *a priori* reason to assume that the relative size of the SET*^GCN4^* sub-population is fixed, and this could potentially vary from species to species and from strain to strain. This is because the magnitude of the extrinsic noise fluctuations may be dependent on multiple (as yet undefined) factors. It is also likely to relate to the growth environment and thus selective forces that, in turn, determine the balance between metabolic burden and enhanced competitiveness. In this context it is important to note other work in yeast showing that rate control of protein synthesis is shared across multiple components of the translation machinery (43). This raises the possibility that heterogeneity in the intracellular abundance of multiple translation apparatus proteins may contribute to the observed SET*^GCN4^* state, perhaps working in synergy with variations in the activity of Gcn2. It would not be surprising to find that such complex functional interactions lie at the root of the type of regulatory heterogeneity we describe here, but their elucidation will require future investigation.

Finally, we note that uORF-mediated posttranscriptional control is also observed in other yeast mRNAs (46) as well as in higher eukaryotes (47). Mammalian and insect (e.g. *Drosophila*) cells possess *ATF4*, a transcription-factor-encoding gene that, analogously to *GCN4*, is subject to uORF-mediated translational regulation (48). It is worth noting that this type of regulatory principle is also thought to apply to other genes, including *ATF5* and *CHOP* (C/EBP homologous protein) (49). The *ATF4* 5’UTR contains two uORFs, and translation of the second of these uORFs, as opposed to translation of the main *ATF4* reading frame, is again subject to modulation by the state of phosphorylation of eIF2α. Remarkably, the *ATF4* regulatory pathway is critical for the survival and proliferation of at least two tumour cell types in response to nutrient deprivation (50). It also plays a role in controlling autophagy (a natural regeneration process that removes defective components in cells) and apoptosis (a process of programmed cell death essential to both the maintenance of homeostasis and tissue growth/development). A striking manifestation of this is that an *ATF4* mutant in *Drosophila* prevents heads from emerging from the thorax during pupation (49).

In conclusion, this study has established the existence of a form of stochastic noise in what appears to be the default operational status of a translational regulatory switch under non-starvation conditions in *S.cerevisiae*. This translational noise is in addition to the transcriptional noise that is generally evident in the expression pathway for all genes. We have determined many of the key features of this novel stochastic system, but future work will need to characterize in detail the full significance of the SET*^GCN4^* expression state in terms of further mechanistic details, the associated fitness trade-offs, and the potential role of such a system in evolutionary terms. Given the existence of multiple genes subject to uORF-mediated translational regulation in eukaryotes, it is possible that related forms of translational stochasticity, and possibly of translational bet-hedging, are operational in at least some of these systems. The mechanisms underpinning such stochastic phenomena may be found to be advantageous for the host solely under certain growth conditions, while close to neutral benefit or disadvantageous in others, so that any overall positive selective value becomes evident only in varying environments. Moving forward from this primary study of the mechanistic basis for gene expression heterogeneity generated by interactions between the translation apparatus and an uORF-containing 5’UTR, future work will need to explore the relationships between this phenomenon and cell viability, stress responses and evolution.

## Supplementary Material

gkad433_Supplemental_File

## Data Availability

Flow Cytometry data generated in this study are available via the FlowRepository website (https://flowrepository.org/id/FR-FCM-Z6ZE). The model presented in this work is available via the EBI BioModels (51) website (MODEL2302230001).
