# Supplementary material for: Stochastic scanning events on the GCN4 mRNA 5’ untranslated region generate cell-to-cell heterogeneity in the yeast nutritional stress response
Source: Nucleic Acids Res. 2023 May 29;51(13):6609–21. doi: 10.1093/nar/gkad433 (PMC10359597; doi:10.1093/nar/gkad433)
Supplement: gkad433_Supplemental_File [file gkad433_supplemental_file.pdf]

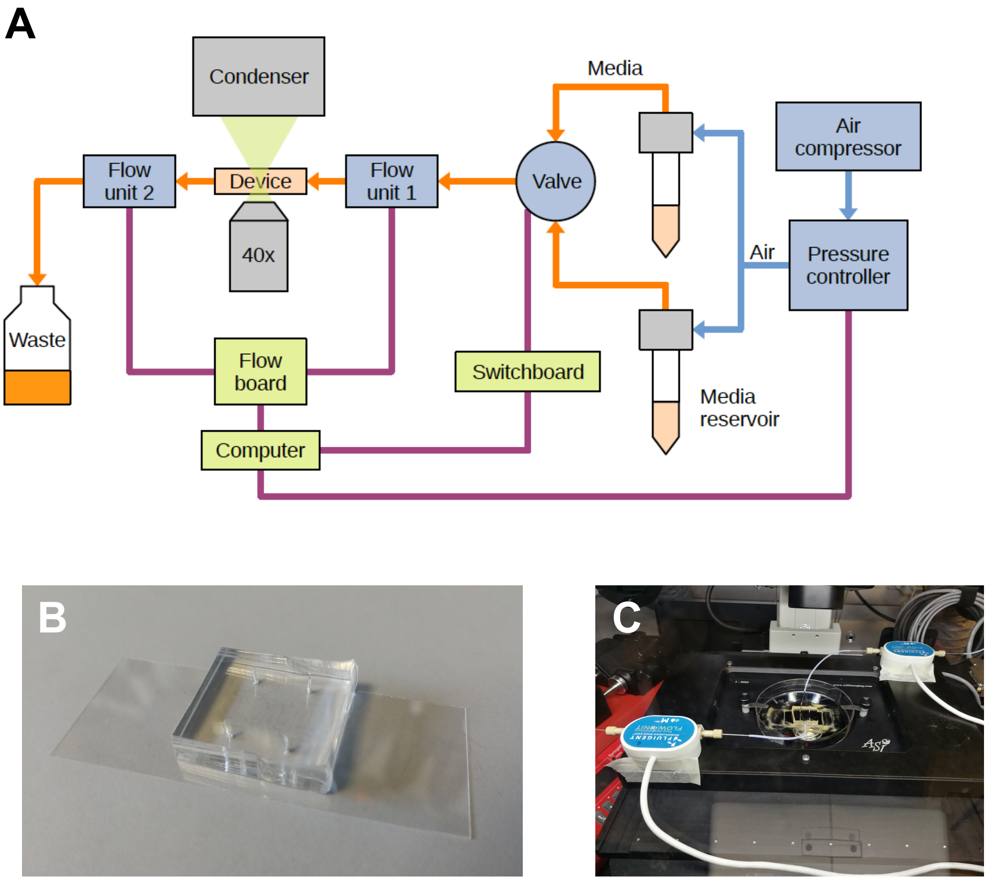

Supp. Figure S1. Microfluidics technology used in this work. Overall design of complete system (**A**), including dual pumps and switching valve system. Microfluidics device, incorporating cell traps (**B**). Same device, mounted on inverted microscope stage (**C**).

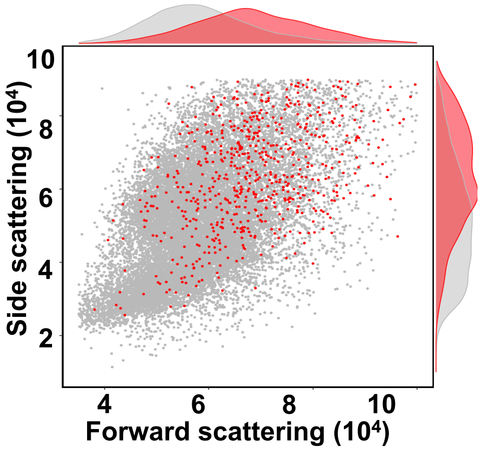

Supp. Figure S2. A version of Figure 3A featuring plots of the intensities of forward and side scattering for the general cell population (grey) and for the SET^{GCN4} subpopulation (red). These data reveal that the SET^{GCN4} state is observed in cells with a wide variety of sizes, shapes and internal structures.

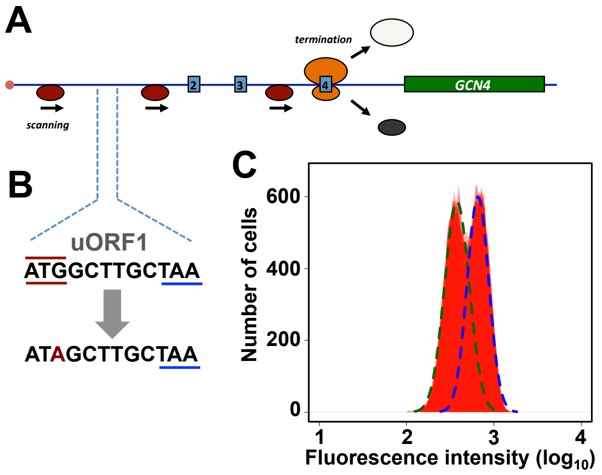

Supp. Figure S3. Single-cell level response to modification of uORF1 in the 5'UTR*GCN4*. Mutation of the uORF1 start codon in the 5'UTR*GCN4* (**A**) to AUA (**B**) leads to a marked change in the response to 3-AT induction. This is illustrated here by the fluorescence distribution after 8 hours of induction, as observed using flow cytometry (**C**). There is a marked reduction in the mean value for reporter fluorescence overall and induction is incomplete. One explanation of this result is that more 43S pre-initiation complexes bypass the uORF2 and uORF3 start codons than would normally scan through uORF1 and continue through to recognise the uORF4 start codon, thus creating an overlapping bimodal distribution [two normal distributions (shown as broken lines) fit the data]. The plots are the result of merged datasets from six biological repeats.

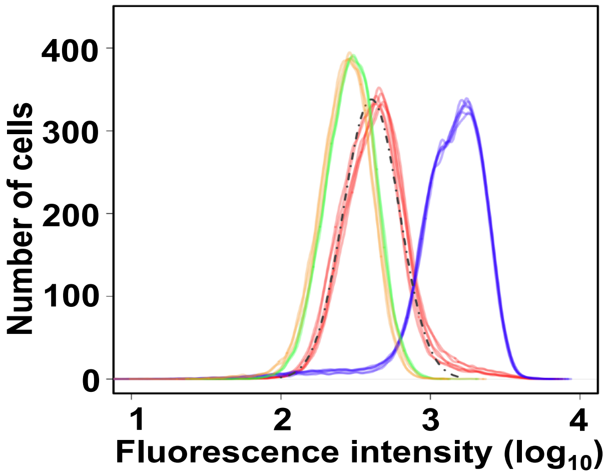

Supp. Figure S4. Combined (overlaid) version of panels A and B from Figure 5 (main text). The outlines in light green and yellow are the same data shown in Figure 5B. The other data are from Figure 5A.

SUPPLEMENTARY SECTION

Mathematical model for modulation of *GCN4* mRNA translation

We consider a model of *GCN4* mRNA translation presented in Figure s1. The model is based on a stochastic kinetic model of translation called the totally asymmetric simple exclusion process (TASEP), which allows multiple ribosomes to translate mRNA concurrently and accounts for their excluded volume interactions (1,2). In the model, the 43S preinitiation complex (PIC) binds to the 5' end of mRNA at rate α. The 43S PIC scans the mRNA for a start codon at speed v. Following translation of uORF1, a fraction η of small ribosomal units (40S) remain on the mRNA and resume the scanning at rate v during which they may acquire a ternary complex (TC) at rate λ. We assume that ribosomes terminating at uORF4 do not resume scanning. In other words, the translation of *GCN4* mRNA in our model occurs only if the scanning 40S does not acquire a TC before reaching the start codon of the uORF4.

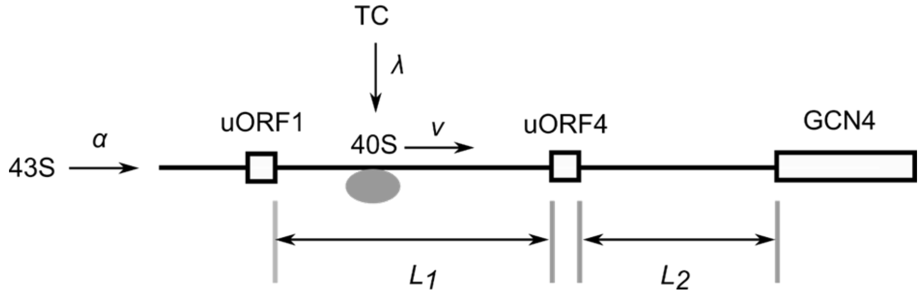

Figure s1: Mathematical model for *GCN4* mRNA translation.

We assume that the rate α at which 43S binds to the 5' end is much lower than the scanning speed v, meaning that the number of 43S, 40S and 40S·TC entities concurrently scanning the transcript is low. This assumption is well justified by polysome-profiling experiments that found only about one ribosome in the 5' UTR under repressing conditions and two ribosomes under de-repressing conditions (3). Under this assumption, the rate of translation of uORF1 is equal to α and the rate at which small ribosomal units pass the uORF1 is $\alpha\eta$. We denote by $P_1(i,t)$ and $P_2(i,t)$ the probabilities that the scanning 40S and 40S·TC are at position i between uORF1 and uORF4, respectively, where $i=1,\dots,L_1$. Under the assumption of low 40S and 40S·TC numbers,

$$\frac{dP_1(1,t)}{dt} = \alpha\eta - (v+\lambda)P_1(1,t), \quad \frac{dP_2(1,t)}{dt} = -vP_2(1,t) + \lambda P_1(1,t),$$

$$\frac{dP_1(i,t)}{dt} = vP_1(i-1,t) - (v+\lambda)P_1(i,t), \quad \frac{dP_2(i,t)}{dt} = vP_2(i-1,t) - vP_2(i,t) + \lambda P_1(i,t), i = 2,\dots,L_1.$$

The solution to these equations in the steady state is

$$P_1(i,t) = \frac{\alpha\eta}{v}\left(\frac{v}{v+\lambda}\right)^i, \quad i = 1,\dots,L_1$$

$$P_2(i,t) = \frac{\alpha\eta}{v}\left[1 - \left(\frac{v}{v+\lambda}\right)^i\right], \quad i = 1,\dots,L_1.$$

The probability that a 40S that resumes scanning after translation of uORF1 reinitiates at uORF4 is equal to

$$P(uORF4) = 1 - \left(v/(v + \lambda)\right)^{L_1}.$$

Next, we consider small ribosomal units that have passed the start codon of uORF4 without acquiring a ternary complex and are scanning for the start codon of *GCN4* codon sequence. We denote by $P_3(i,t)$ and $P_4(i,t)$ the probabilities that the scanning 40S and 40S·TC are at position i between uORF4 and the start codon of *GCN4* coding sequence, respectively, where i=1,…,L_2. The equations for $P_3(i,t)$ and $P_4(i,t)$ are the same as for $P_1(i,t)$ and $P_2(i,t)$ except that L_1 is replaced by L_2, and $\alpha\eta$ is replaced by $vP_1(L_1,t)$, the rate at which a scanning 40S passes the uORF4 without initiation at uORF4. The translation rate of *GCN4* coding sequence is thus equal to

$$k_2 = \eta\alpha\left(\frac{v}{v+\lambda}\right)^{L_1}\left[1 - \left(\frac{v}{v+\lambda}\right)^{L_2}\right].$$

Finally, the probability that a 40S that resumes scanning after translation of uORF1 reinitiates at the start codon of *GCN4* coding sequence is equal to $k_2/(\eta\alpha)$,

$$P(GCN4) = \left(\frac{v}{v+\lambda}\right)^{L_1}\left[1 - \left(\frac{v}{v+\lambda}\right)^{L_2}\right].$$

The model was parameterised based on the available experimental data as follows. The scanning speed of 40S was estimated to be v=10 nt/s *in vitro* (4), but is likely to be higher *in vivo* (and we have therefore also considered the case where v=30 nt/s). The lengths L_1 and L_2 are 198 nt and 139 nt, respectively. The ratio λ/v under repressing and de-repressing conditions, as well as the fraction η of small ribosomal units that resume scanning following translation of uORF1, were initially estimated using previously published expression data (5). There, the probability of reinitiating at uORF4 under repressing conditions was 97%, from which we conclude that λ/v = 0.018. A construct in which the start codons of uORF2, uORF3 and uORF4 were eliminated by mutation reduced *GCN4* mRNA expression by 62% compared to the construct in which all four uORF start codons were removed. From that information, we conclude that η = 62%, because the distance between uORF1 and the *GCN4* coding sequence is long enough for a 40S to be close to certain to acquire the TC. Under de-repressing conditions, the probability of reinitiating at uORF4 was equal to 72%, which gives λ/v = 0.0064. However, in the specific example presented here, we have adjusted the value of λ/v to 0.011 in order to make the model parameters fit the induction data measured by flow cytometry (see Figure 2 in the ***main text***).

The value of α was estimated from the number of ribosomes in the 5' UTR measured by polysome profiling (3), which was one ribosome under repressing conditions. In our model, the number of ribosomes per unit length in nt is equal to α/v in the region between 5' end and uORF1, $\eta\cdot\alpha/v$ in the region between uOTF1 and uORF4, and k_2/v in the region between uORF4 and the start codon of

GCN4 coding sequence. The length from the 5' end to the start codon of uORF1 is L_0=230 nt, and the lengths of the uORF1 and uORF4 are L_{uORF}=12 nt each. This gives

$$\frac{\alpha}{v}=\frac{1}{L_0+L_{uORF}+\eta(L_1+L_{uORF})+\eta\left(\frac{v}{v+\lambda}\right)^{L_1}\left[1-\left(\frac{v}{v+\lambda}\right)^{L_2}\right]L_2}.$$

Parameters and predictions of the model are presented in Table S1. The plots of *P*(uORF4) and *P*(*GCN4*) as a function of *λ/v* are presented in Figure s2. Red-dashed and blue-dot-dashed lines are the TC binding rates under repressing and de-repressing conditions.

Table S1: Parameters and predictions of the *GCN4* mRNA translation model

Parameter	Description	Value	Reference
α	binding rate of 43S to 5' end	$0.026\ s^{-1}$ (*v*=10 nt/s)	model prediction
		$0.078\ s^{-1}$ (*v*=30 nt/s)	
v	assumed scanning speed of 40S and 40S·TC	10 nt/s (*in vitro*)	(4)
		30 nt/s (*in vivo*)	
η	% of ribosomes that resume scanning after terminating at uORF1	62%	(5)
λ/v	ratio of the ternary complex (TC) binding rate and the scanning speed	0.018 (repressing)	model prediction
		0.011 (derepressing)	

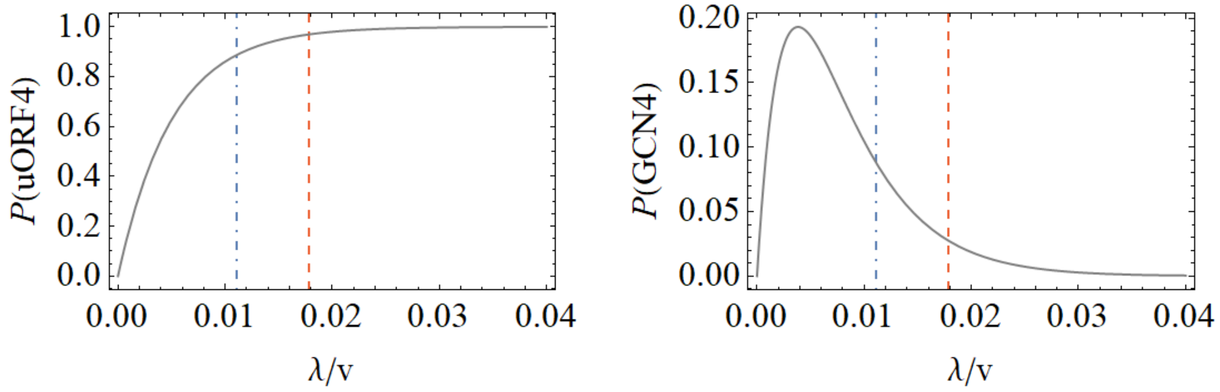

Figure s2: Probabilities P(uORF4) and P(GCN4) for reinitiating at uORF4 and GCN4, respectively. Red dashed and blue dot-dashed lines are values of λ/v under repressing and derepressing conditions, respectively.

Stochastic kinetic model for *GCN4* expression

In the previous section we have explored how the rate of *GCN4* mRNA translation k_2 changes with the TC binding rate. In this section, we provide theoretical arguments supporting the hypothesis that the increased rate of protein synthesis observed in the SET^{GCN4} subpopulation originates from an increased rate of *GCN4* mRNA translation that is comparable to the rate observed under derepressing conditions. To test this hypothesis, we first show that neither the intrinsic noise in the mRNA and protein molecule numbers, nor the extrinsic noise in the transcription rate can explain the origin of the SET^{GCN4} subpopulation.

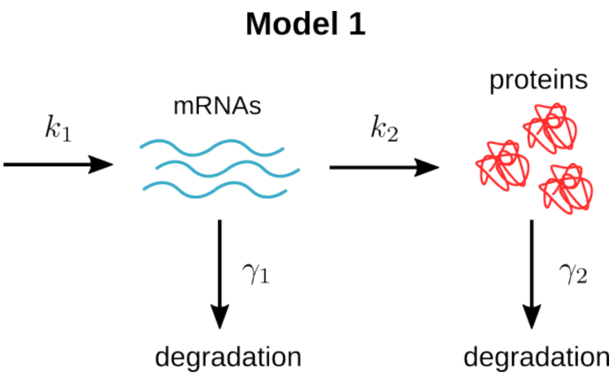

Figure s3: Two-stage model of gene expression from a constitutive promoter.

In the first model, which we refer to as **model 1**, mRNAs are produced at rate k_1 and degraded at rate γ_1, and proteins are produced at rate k_2 and degraded at rate γ_2 (Figure s3). We denote by M the mRNA copy number and by N the protein copy number. In the steady state, the mean mRNA and protein copy numbers are given by, respectively,

$$\langle M \rangle = \frac{k_1}{\gamma_1}, \quad \langle N \rangle = \frac{k_1 k_2}{\gamma_1 \gamma_2},$$

and the variances of M and N are given by

$$\langle M^2 \rangle - \langle M \rangle^2 = \frac{k_1}{\gamma_1}, \quad \langle N^2 \rangle - \langle N \rangle^2 = \frac{k_1 k_2}{\gamma_1 \gamma_2}\left(1 + \frac{k_2}{\gamma_1 + \gamma_2}\right).$$

The coefficient of variation (CV) of the protein copy number,

$$\mathrm{CV} = \sqrt{\frac{\gamma_1 \gamma_2}{k_1}\left(\frac{1}{\gamma_1 + \gamma_2} + \frac{1}{k_2}\right)},$$

decreases under derepressed conditions compared to the repressed conditions, in agreement with the experimental data (Figure 6A in the **main text**). Using the mathematical expression for k_2 computed in the previous section, it follows that the mean *GCN4* protein abundance under steady-state conditions is given by

$$\langle N \rangle = \langle M \rangle \eta \alpha \left(\frac{v}{v + \lambda}\right)^{L_1} \left[1 - \left(\frac{v}{v + \lambda}\right)^{L_2}\right].$$

The rates of mRNA and protein degradation were computed from their respective half-life times, $T_{1/2}$(mRNA) = 19 min (6) and $T_{1/2}$(protein) = 5 min (7). This yielded

$$\gamma_1 = \ln 2 \left(\frac{1}{T_{1/2}(\mathrm{mRNA}) + \frac{1}{90}}\right) = 0.044\ \mathrm{min}^{-1}, \quad \gamma_2 = \ln 2 \left(\frac{1}{T_{1/2}(\mathrm{protein}) + \frac{1}{90}}\right) = 0.15\ \mathrm{min}^{-1},$$

where we have considered the dilution effect attributable to a cell cycle duration of 90 minutes. Since the protein half-life time is much smaller than the cell cycle duration, we do not expect cell-to-cell variations in the cell cycle time to affect the protein degradation rate significantly. However,

they may affect the mRNA degradation rate since this is more comparable to the cell cycle duration.

The mean mRNA copy number was reported to be 26 molecules/cell for the native promoter (8) and 38 molecules/cell for the P_{TEF1} promoter (9). Using the former value, we predicted the transcription rate, and the mean and variance of the protein copy number under repressing and de-repressing conditions for the native promoter, for two scanning speeds, v=10 nt/s and 30 nt/s (Table S2). The value v=10 nt/s derives from an earlier study of translation in a yeast cell-free system (4); v=30 nt/s exemplifies the higher scanning rates that might be achieved in intact yeast cells (*in vivo*; as discussed in reference 4).

Table S2: Parameters and predictions of the model 1 for the native (GCN4) promoter

<table>
<tr><th>Parameter</th><th>Description</th><th colspan="2">Value</th><th>Reference</th></tr>
<tr><td><<i>M</i>></td><td>mean mRNA abundance</td><td colspan="2">26 molecules/cell, CV=20% (native promoter)</td><td>(9)</td></tr>
<tr><td rowspan="3"><<i>N</i>></td><td rowspan="3">mean protein abundance</td><td>v=10 nt/s</td><td>v=30 nt/s</td><td rowspan="3">model prediction</td></tr>
<tr><td>5 molecules/cell, CV=47% (repressing)</td><td>15 molecules/cell, CV=31% (repressing)</td></tr>
<tr><td>16 molecules/cell, CV=30% (derepressing)</td><td>49 molecules/cell, CV=22% (derepressing)</td></tr>
<tr><td>$T_{1/2}$ (mRNA)</td><td>mRNA half-life</td><td colspan="2">19 min</td><td>(7)</td></tr>
<tr><td>$T_{1/2}$ (protein)</td><td>protein half-life</td><td colspan="2">5 min</td><td>(8)</td></tr>
<tr><td>k_1</td><td>transcription rate</td><td colspan="2">1.15 molecules/min</td><td>model prediction</td></tr>
<tr><td rowspan="3">k_2</td><td rowspan="3">translation rate</td><td>v=10 nt/s</td><td>v=30 nt/s</td><td rowspan="3">model prediction</td></tr>
<tr><td>0.029 min^{-1} (repressing)</td><td>0.086 min^{-1} (repressing)</td></tr>
<tr><td>0.092 min^{-1} (derepressing)</td><td>0.27 min^{-1} (derepressing)</td></tr>
<tr><td>γ_1</td><td>mRNA degradation rate</td><td colspan="2">0.044 molecules/min</td><td>model prediction</td></tr>
<tr><td>γ_2</td><td>protein degradation rate</td><td colspan="2">0.15 molecules/min</td><td>model prediction</td></tr>
</table>

Next, we computed the distribution of the protein numbers under repressing and derepressing conditions using an exact solution that was derived in reference (10). To match the experimental data as closely as possible, we modelled transcription from the P_{TEF1} promoter using the value of k_1 = 1.68 min^{-1} derived from the mean mRNA copy number of 38 molecules/cell. The exact protein copy number distribution was then compared to a log-normal distribution, as this type of distribution was observed experimentally (9). The parameters for the log-normal distribution were chosen to match the mean and variance of the protein copy number distribution predicted by the model. The results are presented in Figure s4, showing that the model solution can be well approximated by a log-normal distribution. Importantly, the predicted distribution under normal (repressing) conditions lacks the SET^{GCN4} subpopulation.

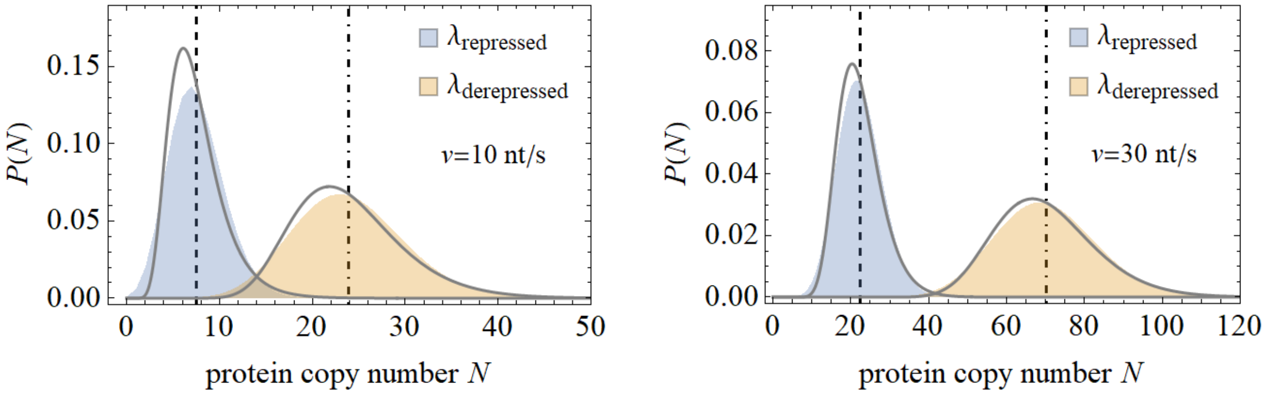

Figure s4: Protein copy number distribution under repressing and derepressing conditions predicted by the model 1. Dashed lines are the corresponding log-normal distributions that match the mean and variance predicted by the model. Vertical dashed lines are the mean protein numbers.

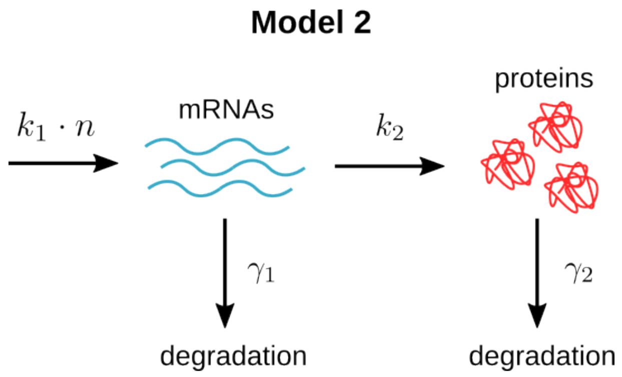

Figure s5: Two-stage model of gene expression from a promoter that produces mRNA in bursts of random size *n*, where *n* is geometrically distributed.

The P_{TEF1} promoter is constitutive. However, for the sake of completeness, we considered a possible scenario in which the SET^{GCN4} subpopulation is somehow generated by fluctuations in the mRNA copy numbers affected by transcriptional bursting (a two-state model). For this purpose, we assumed that mRNAs are produced in bursts of random size *n*, where *n* is geometrically distributed, *n* ~ Geometric(1/(1+b)), and *b* is the mean burst size (Figure s5). What we refer to here as **model 2** is a limiting case of the random telegraph model in which the gene switches between active and inactive states, only producing mRNA from the active state (11). Model 2 is preferred over the random telegraph model because model 2 is known to predict a negative binomial distribution of the mRNA copy number, as experimentally demonstrated previously for the P_{TEF1} promoter (10).

In the steady state, the mean and the variance of the mRNA copy number *M* predicted by model 2 are given by

$$\langle M \rangle = \frac{k_1 b}{\gamma_1}, \quad \langle M^2 \rangle - \langle M \rangle^2 = \frac{k_1 b}{\gamma_1}(1+b).$$

The mean and the standard deviation of *M* were reported to be $\langle M \rangle_{\mathrm{exp}}$ = 38 and σ_{exp} = 11 molecules/cell (10), respectively, from which we predict the values of k_1 = 0.77 min^{-1} and *b* = 2.18.

Using these values, we have computed the protein copy number distribution using stochastic simulations under repressing and derepressing conditions. The resulting distributions were fitted to a log-normal distribution by matching the mean and the variance predicted by model 2 (Figure s6). As before, the predicted distribution under repressing conditions is reasonably well approximated by a log-normal distribution, with no prediction of an enhanced expression sub-population equivalent to that comprising SET^{GCN4} cells.

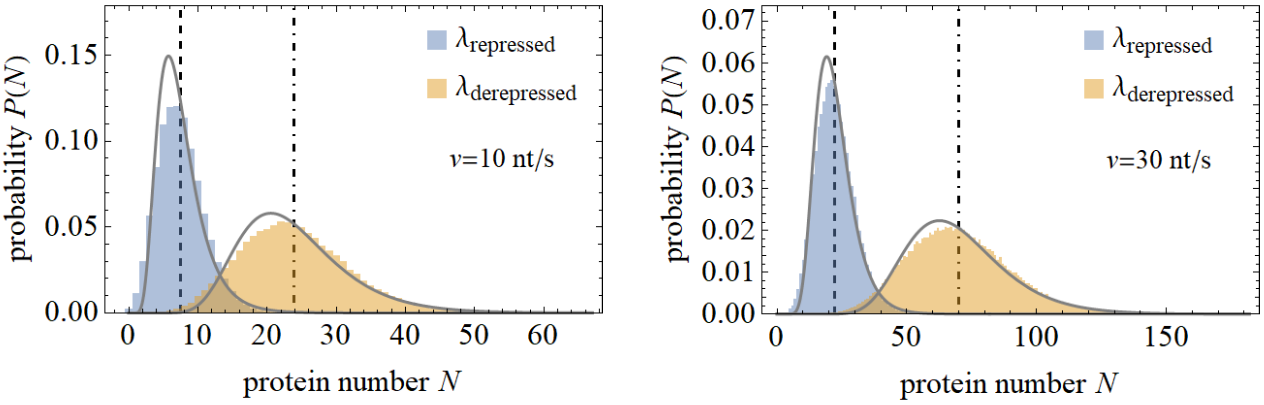

Figure s6: Protein copy number distribution under repressing and derepressing conditions predicted by the model 2. Dashed lines are the corresponding log-normal distributions that match the mean and variance predicted by the model. Vertical dashed lines are the mean protein numbers.

Finally, we considered a possibility that the SET^{GCN4} subpopulation originates from extrinsic noise in the transcription rate. We therefore considered a model similar to model 1, but in which the transcription rate k_1 varies from cell to cell, which we refer to here as **model 3**. We chose a gamma distribution for k_1, because a Poisson distribution of the mRNA copy number predicted by model 1, in which the rate k_1 is gamma distributed, becomes a negative binomial distribution (12). The two parameters of the gamma distribution Gamma(α, β) were computed by matching the mean $\langle M \rangle$ and standard deviation σ_M of the negative binomial distribution with the experimentally measured values of 38 and 11, respectively, according to

$$\alpha = \frac{\langle M \rangle}{\mathrm{FF}_M - 1}, \quad \beta = \frac{1}{\gamma_1} \frac{\mathrm{FF}_M}{\mathrm{FF}_M - 1},$$

where $\mathrm{FF}_M = \sigma_M^2/\langle M \rangle$ = 3.18 is the Fano factor defined by the ratio of the variance and the mean. To obtain the protein copy number distribution for this model, we generated 20 000 values of k_1 from the gamma distribution Gamma(α, β). For each value of k_1, we computed the protein copy number distribution according to the solution of model 1 (10), and from that distribution we generated a random protein copy number N. We grouped all protein copy numbers together and computed their overall distribution. The resulting distribution was then fitted to a log-normal distribution by matching the mean and the variance of the protein copy number predicted by model 3 (Figure s7). Again, no SET^{GCN4} sub-population was predicted under repressing conditions.

In conclusion, the results obtained with models 1, 2 and 3 suggest that neither the intrinsic (molecular) noise in the mRNA and protein copy numbers nor the extrinsic noise in the

transcription rate can account for the SET^{GCN4} subpopulation. Instead, we hypothesise that the origin of the SET^{GCN4} state is due to cell-to-cell fluctuations in the ternary complex binding rate λ caused by cell-to-cell fluctuations in the abundance of Gcn2 kinase.

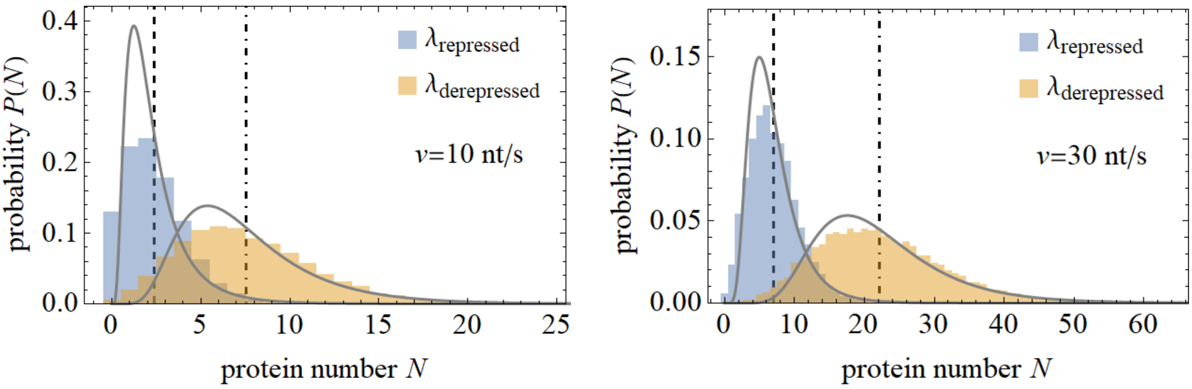

Figure s7: Protein copy number distribution under repressing and derepressing conditions predicted by the model 3. Dashed lines are the corresponding log-normal distributions that match the mean and variance predicted by the model. Vertical dashed lines are the mean protein numbers.

We have reasoned that the hypothetical relationship between Gcn2 kinase activity and the TC binding rate must be a reverse sigmoid function as presented in Figure s8. In other words, a positive fluctuation in the abundance of Gcn2 kinase decreases λ and hence de-represses *GCN4* translation, whereas a negative fluctuation will not further repress *GCN4* translation. A stochastic decrease in Gcn2 activity below the threshold (even to zero) is not expected to repress *GCN4* translation further because once the maximal activity of eIF2 has been attained, the initiation rate of uORF4 cannot increase any further.

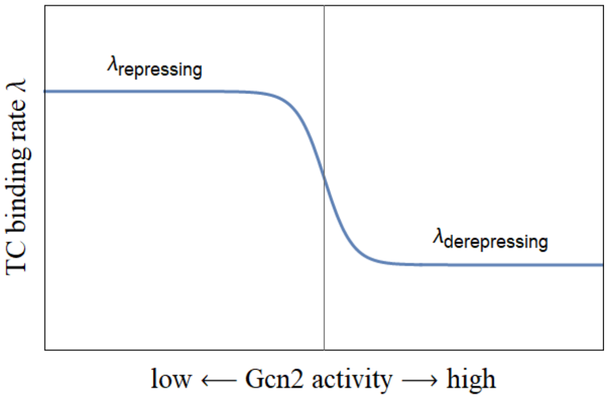

Figure s8: Hypothetical relationship between the TC binding rate and Gcn2 activity.

To simulate this relationship, we generated a set of TC binding rates, each representing one cell, from a log-normal distribution with the mean equal to $\lambda_{repressing}$. For each value of λ, we set $\lambda=\lambda_{repressing}$ if $\lambda>\lambda_t$, and $\lambda=\lambda_{derepressing}$ if $\lambda<\lambda_t$, where $\lambda_t= (\lambda_{derepressing}+\lambda_{repressing})/2$. The CV=11% of the log-normal distribution was selected so that approximately 3% of the values of λ were smaller than λ_t, mimicking the experimental size of the SET^{GCN4} subpopulation. For each value of λ, we generated a protein copy number N from the log-normal distribution parameterised by the mean $\langle N\rangle$ predicted by model 1 for the P_{TEF1} promoter, whereas the CV was set to 34% for the normal state and 27% for the SET^{GCN4} state as measured in the experiment (Figure 6 in the **main text**).

The resulting expression distribution reproducing the SET^{GCN4} state is presented in Figure s9 for two scanning speeds. The molecular mechanism underpinning the hypothetical relationship between the TC binding rate and Gcn2 activity in Figure s8 should be the focus of future investigation.

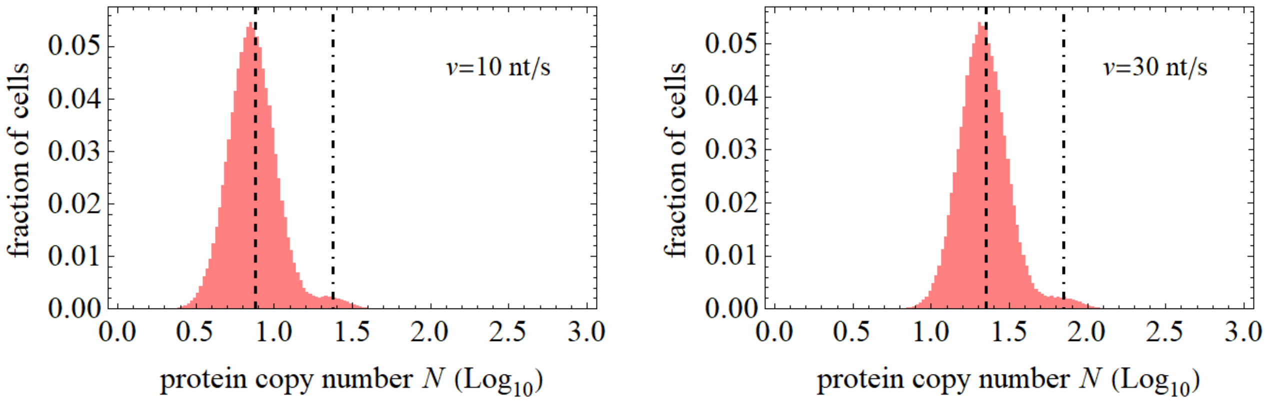

Figure s9: The hypothetical expression distribution of *GCN4* reproducing the SET^{GCN4} population. Dashed and dot-dashed vertical lines correspond to the predicted means <*N*> obtained for $\lambda=\lambda_{repressing}$ and $\lambda=\lambda_{derepressing}$, respectively.

Statistical analysis of the fluorescence intensity distributions

In the previous section, we developed a kinetic model of *GCN4* mRNA expression that gave a plausible explanation for the origin of the SET^{GCN4} state. In this section, we give statistical, ***model-free*** evidence that the experimentally measured protein copy number distribution is best described by a mixture of two distributions rather than a single distribution.

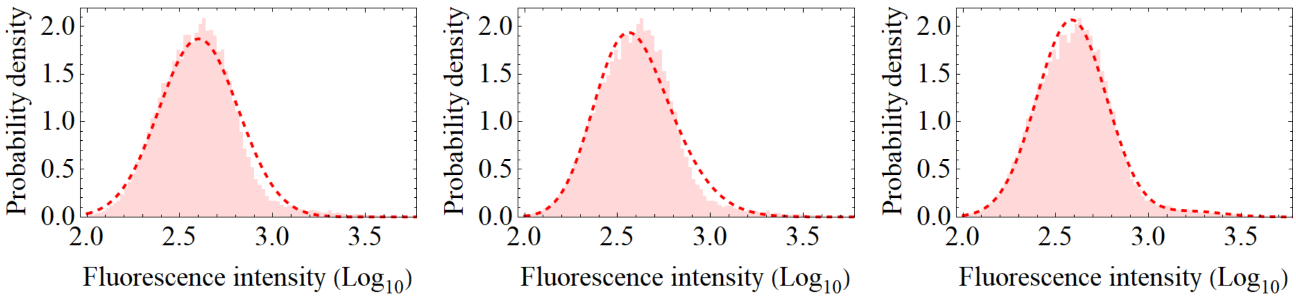

Figure s10: Fluorescence intensity data from Figure 2C in the main text fitted to various distributions. Left: the normal distribution. Middle: the skew normal distribution. Right: a mixture of two normal distributions.

Figure s10 shows the fluorescence intensity data replotted from Figure 2C in the **main text** and fitted to the normal distribution, the skew normal distribution and ***a combination*** of two normal distributions. We chose the skew normal distribution over the negative binomial distribution as our choice of a skewed distribution, because the variance of our data is smaller than the mean, whereas for the negative binomial distribution the variance is always larger than the mean. As expected, neither the normal nor the skewed normal distribution fits the data very well. Instead, a mixture of two normal distributions is found to fit the data significantly better. The best fit was found for the mixture weight of 2.5% for the SET^{GCN4} subpopulation. In contrast, the fluorescence intensity data from strains that lacked the SET^{GCN4} subpopulation fit well to a single normal distribution (Figure s11).

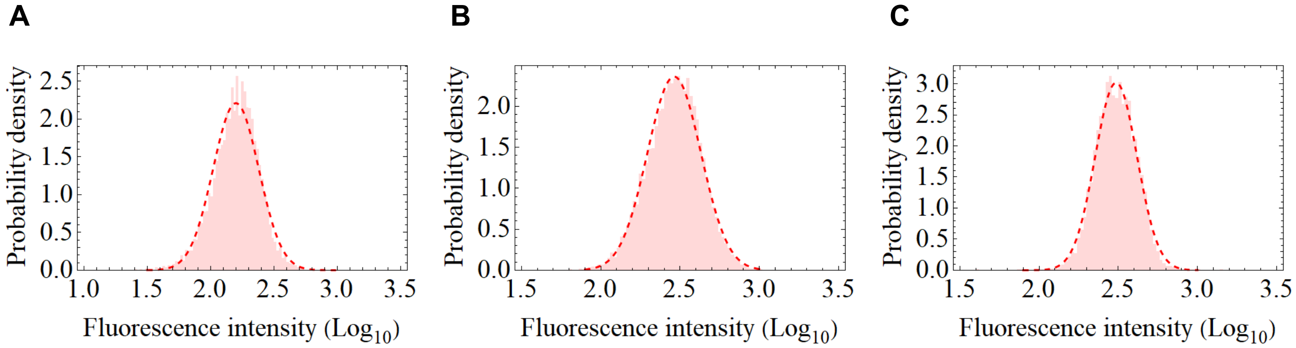

Figure s11: Fluorescence intensity data from Figures 4, 5B and 5C in the main text fitted to the normal distribution. (**A**) A strain with a mutation in the uORF1 ATG start codon to ATA; (**B**) A strain lacking Gcn2 kinase; (**C**) A strain with a Ser51Ala mutation in eIF2α.

Moreover, a single normal distribution provided an equally good fit to data generated by a strain carrying a control construct in which the 5'UTR^{GCN4} of the main experimental construct described in Figure 2A (**main text**) was replaced by a short (30nt) unstructured 5'UTR (Figure s12).

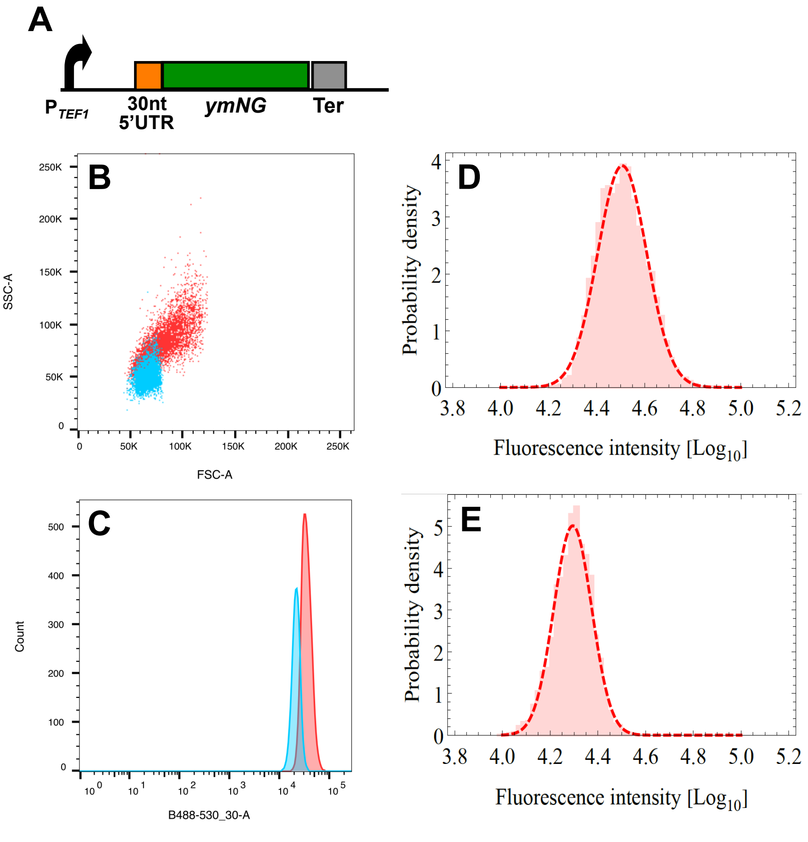

Figure s12: A non-structured 5'UTR does not generate a SET^{GCN4} sub-population. (A) The control expression construct with an unstructured 30nt 5'UTR integrated into the yeast genome. **(B)** Forward scattering height and area values were used to discriminate between singlet and doublet cell populations observed in flow cytometry of the strain bearing the genome-integrated control expression construct. **(C)** Plots of flow cytometry fluorescence intensity (log_{10}) vs cell-count for the doublet (red) and singlet (blue) subpopulations, respectively. Further analysis reveals a good fit to a normal distribution for both the doublets **(D)** and for the singlets **(E)**, thus confirming the absence of SET^{GCN4} cells in either part of the cell population.

We investigated whether the technical issue of detection events associated with what are referred to as 'doublets' in flow cytometry, in which two cells simultaneously (or, in the case of *S.cerevisiae*, single cells in a significantly progressed state of budding) pass through the detector to create one

detection event, could influence the observed fluorescence intensity distributions. Discrimination of doublets and singlets was performed using a standard procedure based on scattering data (Figure 12B), allowing us to investigate the fluorescence intensity distributions for doublets and singlets separately (Figure 12C). Both the doublet and singlet data gave good fits to the normal distribution (Figure 12D,E).

References (Supplementary Data section)

(1) MacDonald, C.T., Gibbs, J.H. and Pipkin, A.C. (1968) Kinetics of biopolymerization on nucleic acid templates. *Biopolymers* **6**, 1-25.

(2) You, T., Stansfield, I., Romano, M.C. *et al.* (2011) Analysing *GCN4* translational control in yeast by stochastic chemical kinetics modelling and simulation. *BMC Syst. Biol.* **5**, 131.

(3) Arava Y., Boas, F. E., Brown, O. P. and Herschlag D. (2005) Dissecting eukaryotic translation and its control by ribosome density mapping. *Nucleic Acids Res.* **33**, 2421-32.

(4) Berthelot, K., Muldoon, M., Rajkowitsch, L., Hughes, J. and McCarthy, J.E.G. (2004) Dynamics and processivity of 40S ribosome scanning on mRNA in yeast. *Molec. Microbiol.* **51**, 987-1001.

(5) Grant C.M., Miller P.F. and Hinnebusch A. G. (1994) Requirements for intercistronic distance and level of eukaryotic initiation factor 2 activity in reinitiation on GCN4 mRNA vary with the downstream cistron. *Mol. Cell. Biol.* **14**, 2616-28.

(6) Geisberg J.V., Moqtaderi Z., Fan X., Ozsolak F. and Struhl K. (2014) Global Analysis of mRNA Isoform Half-Lives Reveals Stabilizing and Destabilizing Elements in Yeast. *Cell* **156**, 812-824.

(7) Belle A., Tanay A., Bitincka L., Shamir R. and O'Shea E. K. (2006) Quantification of protein half-lives in the budding yeast proteome. *Proc. Natl. Acad. Sci.* **103**, 13004-9.

(8) Pelechano V., Chávez S. and Pérez-Ortín J.E. (2010) A Complete Set of Nascent Transcription Rates for Yeast Genes. *PLoS ONE* **5**, e15442.

(9) Dacheux E., Malys N., Meng X., Ramachandran V., Mendes P. and McCarthy J. E. G. (2017) Translation initiation events on structured eukaryotic mRNAs generate gene expression noise. *Nucleic Acids Research* **45**(11), 6981-6992.

(10) Bokes, P., King, J.R., Wood, A.T.A. *et al.* (2012) Exact and approximate distributions of protein and mRNA levels in the low-copy regime of gene expression. *J. Math. Biol.* **64**, 829-854.

(11) Shahrezaei V. and Swain P. S. (2008) Analytical distributions for stochastic gene expression. *Proc. Natl. Acad. Sci.* **105**(11), 17256-17261.

(12) Ham L., Jackson M. and Stumpf M. P. H. (2021) Pathway dynamics can delineate the sources of transcriptional noise in gene expression. *eLife* **10**, e69324.
